# Supplementary material for: Potential Infection Risks of the Wheat Stripe Rust and Stem Rust Pathogens on Barberry in Asia and Southeastern Europe
Source: Plants (Basel). 2021 May 11;10(5):957. doi: 10.3390/plants10050957 (PMC8151100; doi:10.3390/plants10050957)
Supplement: Supplementary file 1 [file plants-10-00957-s001.zip › plants-1187303-supplementary.pdf]

Table S1. Elevations, latitudes, longitudes, monthly infection index (MINF), Fav-month (F) and infection risk (MINF x F) values of 607 locations for the stripe rust pathogen, *Puccinia striii* *P. graminis* f. sp. *tritici* (*Pgt*) in relation to barberry growth in Asia and adjoining European region

						Pst				
Region and Country	Location	Code	Elevation (m)	Latitude (°N)	Longitude (°E)	MINF (A)	Fav-month (F)	Favourable index (A x F)	Risk score	Month
**East Asia (250)**										
Bangladesh	Nalian Range, BD	BD1	4.2	22.1986	89.4880	0.0000	0	0.0000	0	0
Bangladesh	Rangamati, BD	BD2	546.6	25.0170	91.2898	0.0041	0.33	0.0014	0	4
Bangladesh	Genduram, BD	BD3	82.3	25.5335	89.6638	0.0000	0	0.0000	0	0
Bangladesh	Durgaour, BD	BD4	6.6	22.9695	91.1579	0.0000	0	0.0000	0	0
Bangladesh	Ati Ulain, BD	BD5	9.8	23.9772	89.8835	0.0000	0	0.0000	0	0
Bhutan	Wangdue Phodrang, BH	BH1	3903.8	27.6991	90.2955	0.2095	0.33	0.0691	1	4
Bhutan	Samdrup Jongkhar, BH	BH2	618.9	26.9035	91.1909	0.0067	0.17	0.0011	0	2
Bhutan	Gasa, BH	BH3	3911.5	27.8789	89.9055	0.0913	0.25	0.0228	1	3
Bhutan	Lhuntse, BH	BH4	3305.8	27.7332	91.2348	0.0229	0.25	0.0057	0	3
Bhutan	Kangpar, BH	BH5	2178.5	27.2166	91.7842	0.0501	0.17	0.0085	1	2
Bhutan	Xigate, BH	BH6	3924.5	27.5190	88.8948	0.1439	0.25	0.0360	1	3
Bhutan	Putao 2, BH	BH7	2533.8	27.0112	98.2134	0.1087	0.25	0.0272	1	3
Bhutan	Putao 3, BH	BH8	926.5	26.7859	98.7077	0.0141	0.17	0.0024	0	2
Bhutan	Daifam, BH	BH9	631.3	26.9427	92.0698	0.0145	0.25	0.0036	0	3
China	Litang, Sichuan, CH	CH1	4184.1	29.4684	100.4476	0.0130	0.17	0.0022	0	2
China	Batang 1, Sichuan, CH	CH2	3958.5	29.2778	99.3682	0.0172	0.25	0.0043	0	3
China	Kangding, Sichuan, CH	CH3	3980.7	29.5066	101.8538	0.0112	0.33	0.0037	0	4
China	Shawan 1, Sichuan, CH	CH4	774.9	29.2002	103.6116	0.2691	0.67	0.1803	2	8
China	Suijiang, Yunnan, CH	CH5	1176.4	28.5460	103.8753	0.2851	0.83	0.2366	2	10
China	Dechang, Sichuan, CH	CH6	2238.6	27.1863	102.2933	0.0599	0.17	0.0102	1	2
China	Huangling, Shaanxi, CH	CH7	1307.4	35.6127	108.7532	0.0465	0.17	0.0079	0	2
China	Jiaokou, Shaanxi, CH	CH8	1289.2	37.0289	111.2581	0.0254	0.17	0.0043	0	2
China	Shennonhjia, Hubei, CH	CH9	1056.5	31.7750	110.6868	0.0236	0.25	0.0059	0	3
China	Kaixian, Chongqing, CH	CH10	731.7	31.4006	108.2698	0.0963	0.58	0.0559	1	7
China	Hejiang, Sichuan, CH	CH11	538	28.6618	105.6331	0.2081	0.58	0.1207	2	7
China	Alxa, Inner Mongolia, CH	CH12	1888.9	38.9339	101.5023	0.0293	0.17	0.0050	0	2

Region and Country	Location	Code	Elevation (m)	Latitude (°N)	Longitude (°E)	*Pst* MINF (A)	Fav-month (F)	Favourable index (A x F)	Risk score	Month
China	Tianzhu, Gansu, CH	CH13	2836.8	37.3091	102.7327	0.0121	0.25	0.0030	0	3
China	Guinan, Qinghai, CH	CH14	3405.9	35.5412	101.2825	0.0000	0	0.0000	0	0
China	Nanzhao, Henan, CH	CH15	388.9	33.3306	112.3567	0.0143	0.33	0.0047	0	4
China	Zhenxiong, Yunnan, CH	CH16	1272.1	27.5376	105.1497	0.1846	0.58	0.1071	2	7
China	Fengqing, Yunnan, CH	CH17	1666.8	24.4972	99.9642	0.0274	0.33	0.0091	1	4
China	Batang 2, Sichuan, CH	CH18	3958.5	29.1618	99.3050	0.0172	0.25	0.0043	0	3
China	Daguan, Yunnan, CH	CH19	1029.5	28.0818	104.0071	0.2235	0.67	0.1497	2	8
China	Danba, Sichuan, CH	CH20	3756.5	30.9117	101.9857	0.0050	0.08	0.0004	0	1
China	Dayi, Sichuan, CH	CH21	2073.4	30.5632	103.4297	0.3144	0.58	0.1824	2	7
China	Shawan 2, Sicuan, CH	CH22	774.9	29.3065	103.5176	0.2691	0.67	0.1803	2	8
China	Junlian xian, Sichuan, CH	CH23	1029.5	28.0342	104.4404	0.2235	0.67	0.1497	2	8
China	Banan, Chongqing, CH	CH24	604.5	29.3832	106.8574	0.1795	0.58	0.1041	2	7
China	Xixia, Henan, CH	CH25	571.3	33.4690	111.2969	0.0205	0.25	0.0051	0	3
China	Taibai, Shaanxi, CH	CH26	1407.4	33.8714	107.6924	0.0648	0.08	0.0052	0	1
China	Wangyi, Shaanxi, CH	CH27	923.2	35.0669	109.1426	0.0501	0.17	0.0085	1	2
China	Maiji, Gansu, CH	CH28	1547.8	34.1628	106.4619	0.0330	0.17	0.0056	0	2
China	Zhenba, Shaanxi, CH	CH29	1038.5	32.4366	107.7363	0.0795	0.42	0.0334	1	5
China	Tewo, Gansu, CH	CH30	3180.8	33.9443	103.8691	0.0000	0	0.0000	0	0
China	Jiuzhi,Qinghai, CH	CH31	4029.8	33.3223	101.4521	0.0000	0	0.0000	0	0
China	Wudu, Gansu, CH	CH32	1632.2	33.4324	105.0556	0.0389	0.08	0.0031	0	1
China	Jiulong,Sichuan, CH	CH33	3441.7	28.8072	101.6279	0.0667	0.42	0.0280	1	5
China	Xinlong, Sichuan, CH	CH34	4386.6	30.6790	99.9497	0.0000	0	0.0000	0	0
China	Yanyuan, Sichuan, CH	CH35	2585.9	27.5775	101.6643	0.0510	0.25	0.0128	1	3
China	Huize, Yunnan, CH	CH36	2176.1	26.6681	103.6089	0.0308	0.25	0.0077	0	3
China	Jinsha, Guizhou, CH	CH37	1089.4	27.4313	106.5203	0.1127	0.5	0.0564	1	6
China	Mohe, Heilongjiang, CH	CH38	708.8	52.7868	121.8757	0.0548	0.42	0.0230	1	5
China	Jianning, Fujian, CH	CH39	463.6	26.6607	116.8055	0.1149	0.25	0.0287	1	3
China	Yuxian, Yunnan, CH	CH40	1666.8	24.2405	99.9745	0.0274	0.33	0.0091	1	4
China	Putao 4, Mym	CH41	1195.3	26.6670	97.8651	0.0145	0.25	0.0036	0	3

Region and Country	Location	Code	Elevation (m)	Latitude (°N)	Longitude (°E)	*Pst* MINF (A)	Fav-month (F)	Favourable index (A x F)	Risk score	Month
China	Maizhokunggar, Lhasa, CH	CH42	4832.9	30.0701	91.7677	0.0000	0	0.0000	0	0
China	Dengqen, Tibet, CH	CH43	4781.1	32.1115	95.1405	0.0000	0	0.0000	0	0
China	Sunan, Gansu, CH	CH44	3684.6	38.3684	99.9525	0.0000	0	0.0000	0	0
China	Tahe, Heilongjiang, CH	CH45	451.9	52.4952	125.4573	0.0571	0.33	0.0188	1	4
China	Yakeshi, Inner Mongolis, CH	CH46	907.6	50.6206	121.7274	0.0083	0.17	0.0014	0	2
China	Jinshantun, Heilongjiang, CH	CH47	411.1	47.3042	129.4618	0.0050	0.17	0.0009	0	2
China	Zalantun, Inner Mongolia, CH	CH48	923.3	47.8674	121.3319	0.0034	0.08	0.0003	0	1
China	Zamtang, Sicuana, CH	CH49	4088.9	31.6847	101.2049	0.0000	0	0.0000	0	0
China	Wuxi, Chongqing, CH	CH50	1353	31.6099	109.4227	0.0485	0.33	0.0160	1	4
China	Yongding, Huanan, CH	CH51	590.2	29.3377	110.2577	0.1299	0.58	0.0753	1	7
China	Xiushui, Jiangxi, CH	CH52	302.9	29.0307	114.3446	0.1081	0.58	0.0627	1	7
China	Yingde, Guangdong, CH	CH53	291.3	24.4007	113.6415	0.0982	0.17	0.0167	1	2
China	Tian'e, Guangxi, CH	CH54	791.7	24.8002	106.7420	0.0809	0.33	0.0267	1	4
China	Baiyu, Sichuan, CH	CH55	4334.3	31.2419	99.5625	0.0000	0	0.0000	0	0
China	Alxa Zuoqi, Inner Mongolia, CH	CH56	1331.6	39.2672	104.4844	0.0023	0.08	0.0002	0	1
China	Biru, Tibet, CH	CH57	4912.3	30.9927	93.9485	0.0000	0	0.0000	0	0
China	Nagqu 1, Tibet, CH	CH58	4891.6	31.4062	92.6521	0.0000	0	0.0000	0	0
China	Nagqu 2, Tibet, CH	CH59	4831.4	31.1151	91.6084	0.0000	0	0.0000	0	0
China	Gyaca 1, Tibet, CH	CH60	4702.1	29.2203	92.8993	0.0015	0.08	0.0001	0	1
China	Nangxian, Tibet, CH	CH61	4089.9	28.7869	93.2305	0.0168	0.17	0.0029	0	2
China	Jiulong, Sichuan, CH	CH62	3779.8	28.6337	101.4741	0.0503	0.33	0.0166	1	4
China	Wenxian, Gansu, CH	CH63	1872.1	32.7882	104.5942	0.0485	0.17	0.0082	1	2
China	Wenchuan, Sichuan, CH	CH64	2889.9	31.1668	103.2319	0.1251	0.42	0.0525	1	5
China	Barkam, Sichuan, CH	CH65	3745.5	32.0835	101.9575	0.0000	0	0.0000	0	0
China	Hongya, Sichuan,CH	CH66	1088.4	29.5075	103.0506	0.3338	0.83	0.2771	2	10
China	Hanyuan, Sichuan, CH	CH67	2864.4	29.6174	102.3805	0.1449	0.5	0.0725	1	6
China	Butuo 1, Sichuan, CH	CH68	3953.2	29.2443	101.8916	0.0312	0.33	0.0103	1	4
China	Butuo 2, Sichuan, CH	CH69	2372.5	27.6359	102.8913	0.0769	0.42	0.0323	1	5
China	Muchuan, Sichuan, CH	CH70	1176.4	28.8360	103.9021	0.2851	0.83	0.2366	2	10

Region and Country	Location	Code	Elevation (m)	Latitude (°N)	Longitude (°E)	*Pst* MINF (A)	Fav-month (F)	Favourable index (A x F)	Risk score	Month
China	Leibo, Sichuan, CH	CH71	1567.4	28.3199	103.7043	0.2536	0.58	0.1471	2	7
China	Luquan, Yunnan, CH	CH72	1987.5	25.9146	102.3860	0.0158	0.08	0.0013	0	1
China	Lanping, Yunnan, CH	CH73	2739	26.5797	99.2494	0.0902	0.33	0.0298	1	4
China	Huili, Sichuan, CH	CH74	2026.9	26.7859	102.4629	0.0399	0.17	0.0068	0	2
China	Dayao, Yunnan, CH	CH75	2039.1	26.0183	101.1390	0.0250	0.25	0.0063	0	3
China	Mainling, Tibet, CH	CH76	3752.1	29.3401	94.2012	0.0328	0.33	0.0108	1	4
China	Jinchuan, Sichuan, CH	CH77	3737.3	31.1386	102.0234	0.0052	0.17	0.0009	0	2
China	Zhuanglang, Gansu, CH	CH78	1817.4	35.1927	105.8686	0.0278	0.25	0.0069	0	3
China	Zhenyuan, Gansu, CH	CH79	1366.9	35.8989	107.1321	0.0460	0.17	0.0078	0	2
China	Dingbian, Shaanxi, CH	CH80	1508.9	37.0522	107.5646	0.0288	0.17	0.0049	0	2
China	Qilian, Qinghai, CH	CH81	3572.4	37.7542	100.7929	0.0043	0.08	0.0003	0	1
China	Nanbu, Sichuan, CH	CH82	448.7	31.2419	105.8906	0.0930	0.25	0.0233	1	3
China	Ulan 2, Qinghai, CH	CH83	3683.4	37.0561	98.3320	0.0018	0.08	0.0001	0	1
China	Jomdo, Tibet, CH	CH84	4398.9	32.2881	97.4531	0.0000	0	0.0000	0	0
China	Wenchuan 2, Sichuan, CH	CH85	2576.4	31.5115	103.5824	0.1991	0.33	0.0657	1	4
China	Pengzhou, Sichuan, CH	CH86	1759.8	31.0956	103.8605	0.3798	0.58	0.2203	2	7
China	Mianzhu, Sichuan, CH	CH87	979.2	31.3992	104.0419	0.1939	0.58	0.1124	2	7
China	Pingshan 2, Sichuan, CH	CH88	671.2	28.8144	104.2509	0.1846	0.67	0.1237	2	8
China	Suijiang 2, Yunnan, CH	CH89	1176.4	28.6144	103.9131	0.2851	0.83	0.2366	2	10
China	Shuifu, Yunan, CH	CH90	1029.5	28.4600	104.2207	0.2235	0.67	0.1497	2	8
China	Tewo 2, Gansu, CH	CH91	3180.8	33.9802	103.5977	0.0000	0	0.0000	0	0
China	Meigu 2, Sichuan, CH	CH92	1934.7	28.7679	103.1637	0.2241	0.58	0.1300	2	7
China	Ebian, Sichuan, CH	CH93	1491.2	29.0947	103.4239	0.2970	0.67	0.1990	2	8
China	Mabian, Sichuan, CH	CH94	1934.7	28.7946	103.3180	0.2241	0.58	0.1300	2	7
China	Wenchuan 3, Sichuan, CH	CH95	2073.4	30.9288	103.2875	0.3144	0.58	0.1824	2	7
China	Ge'gyai, Tibet, CH	CH96	5200.6	31.2560	82.7681	0.0000	0	0.0000	0	0
China	Karakax, Xinjiang, CH	CH97	1227.1	38.2865	79.8238	0.0026	0.08	0.0002	0	1
China	Hoboksar, Xinjiang, CH	CH98	500.1	45.5956	87.0308	0.0123	0.17	0.0021	0	2
China	Hejing, Xinjiang, CH	CH99	2770.2	42.7599	86.5035	0.0000	0	0.0000	0	0

Region and Country	Location	Code	Elevation (m)	Latitude (°N)	Longitude (°E)	*Pst* MINF (A)	Fav-month (F)	Favourable index (A x F)	Risk score	Month
China	Kumul, Xinjiang, CH	CH100	1427.4	41.3900	94.0620	0.0000	0	0.0000	0	0
China	Ruoqiang, Xinjiang, CH	CH101	3060.9	38.8363	90.3706	0.0086	0.08	0.0007	0	1
China	Zhidoi, Qinghai, CH	CH102	4975.1	35.3317	91.3374	0.0000	0	0.0000	0	0
China	Shuanghu, Tibet, CH	CH103	4792.1	32.2648	90.3706	0.0000	0	0.0000	0	0
China	Zhengxiangbaiqi, Inner Mongolia, CH	CH104	1201.9	42.8244	114.7163	0.0000	0	0.0000	0	0
China	Urad Houqi, Inner Mongolia, CH	CH105	1454.1	41.3900	106.7183	0.0000	0	0.0000	0	0
China	Aba, Sichuan, CH	CH106	3830.1	31.8975	102.2082	0.0379	0.17	0.0064	0	2
China	Baoji2, Shaanxi, CH	CH107	1368.9	34.3787	107.1298	0.0853	0.33	0.0282	1	4
China	Bazhong, Sichuan, CH	CH108	597.9	31.8666	106.7181	0.1362	0.58	0.0790	1	7
China	Chengxian, Gansu, CH	CH109	1489.3	33.7436	105.6880	0.0703	0.33	0.0232	1	4
China	Dali, Yunan, CH	CH110	2212.9	25.6058	100.1786	0.0489	0.17	0.0083	1	2
China	Degen, Yunan, CH	CH111	1714.4	24.1786	100.7496	0.0206	0.17	0.0035	0	2
China	Deyang, Sichuan, CH	CH112	979.2	31.1405	104.3301	0.2742	0.67	0.1837	2	8
China	Dinxi, Gansu, CH	CH113	1982.8	35.5888	104.6410	0.0133	0.17	0.0023	0	2
China	Fengxian, Shaanxi, CH	CH114	1444.4	33.9258	106.5501	0.0982	0.33	0.0324	1	4
China	Gnagu, Gansu, CH	CH115	1852.8	34.7232	105.3472	0.0380	0.25	0.0095	1	3
China	Gannan, Gansu, CH	CH116	2022.9	36.4891	103.6229	0.0018	0.08	0.0001	0	1
China	Ganzi, Sichuan, CH	CH117	3897.4	30.0683	101.9874	0.0391	0.25	0.0098	1	3
China	Guangyuan, Sichuan, CH	CH118	845.9	32.4557	105.4557	0.1336	0.58	0.0775	1	7
China	Haixi, Qinghai, CH	CH119	3362.6	37.3859	97.3790	0.0081	0.08	0.0007	0	1
China	Hanzong, Shaanxi, CH	CH120	1140.6	33.0695	106.9625	0.1791	0.58	0.1039	2	7
China	Huixian, Gansu, CH	CH121	1402.6	33.7688	106.0569	0.0749	0.33	0.0247	1	4
China	Lueyang, Shaanxi, CH	CH122	1249.6	34.6303	107.1306	0.0606	0.42	0.0255	1	5
China	Kunming, Yunan, CH	CH123	1938.5	24.9522	102.8935	0.0202	0.08	0.0016	0	1
China	Lanzhou, Gansu, CH	CH124	2022.9	36.4891	103.6229	0.0018	0.08	0.0001	0	1
China	Linagdang, Gansu, CH	CH125	1402.6	33.9050	106.2660	0.2618	0.33	0.0864	1	4
China	Liangshun, Sichuan, CH	CH126	2438.1	27.8584	102.2765	0.0434	0.25	0.0108	1	3
China	Lijiang, Yunan, CH	CH127	2618.8	26.8624	100.1740	0.0387	0.17	0.0066	0	2
China	Linxia, Gansu, CH	CH128	2270.8	35.5903	103.2469	0.0168	0.25	0.0042	0	3

Region and Country	Location	Code	Elevation (m)	Latitude (°N)	Longitude (°E)	*Pst* MINF (A)	Fav-month (F)	Favourable index (A x F)	Risk score	Month
China	Lixian, Gansu, CH	CH129	1852.8	34.7232	105.3472	0.0380	0.25	0.0095	1	3
China	Longnan, Gansu, CH	CH130	2070.6	33.3986	104.9376	0.0992	0.42	0.0417	1	5
China	Maijigen, Gansu, CH	CH131	1547.8	34.3881	106.0023	0.0623	0.33	0.0205	1	4
China	Mianyang, Sichuan, CH	CH132	601.3	31.4811	104.6458	0.1521	0.58	0.0882	1	7
China	Panlogjhen, Shaanxi, CH	CH133	1368.9	34.4162	107.2039	0.0853	0.33	0.0282	1	4
China	Panzihua, Sichuan, CH	CH134	1901.9	26.6374	101.7038	0.0385	0.08	0.0031	0	1
China	Qiangyang2, Sichuan, CH	CH135	1249.6	34.6281	107.0939	0.0606	0.42	0.0255	1	5
China	Qingshui, Gansu, CH	CH136	1720.2	34.7068	106.1763	0.0558	0.33	0.0184	1	4
China	Shannan, Tibet, CH	CH137	4597.2	29.2622	91.7658	0.0000	0	0.0000	0	0
China	Taibai2, Shaanxi, CH	CH138	1368.9	34.0594	107.3061	0.0853	0.33	0.0282	1	4
China	Tianshui, Gansu, CH	CH139	1671.9	34.5621	105.7233	0.0435	0.33	0.0143	1	4
China	Wangchuamjen, Gansu, CH	CH140	1583.9	34.2247	105.5837	0.0635	0.33	0.0210	1	4
China	Yaan, Sichuan, CH	CH141	1765.3	29.9757	102.9928	0.4467	0.67	0.2993	2	8
China	Yuxi, Yunan	CH142	1713.7	24.3535	102.4571	0.0172	0.17	0.0029	0	2
India	Deema Range, Uttarakhand, IN	IN1	891.9	29.6318	78.8533	0.0062	0.17	0.0011	0	2
India	Champawat,UK	IN2	1445.4	29.0187	80.6770	0.0703	0.33	0.0232	1	4
India	Lunglei, Mizoram,IN	IN3	555.2	22.9518	92.9432	0.0017	0.17	0.0003	0	2
India	Devidhura Range, Uttarakhand, IN	IN4	864.4	29.2682	79.9737	0.0116	0.33	0.0038	0	4
India	Kheti, Uttarakhand, IN	IN5	2163.9	29.9083	80.1387	0.1587	0.25	0.0397	1	3
India	Birgola, Uttarakhand, IN	IN6	1116.5	29.2120	80.2774	0.0450	0.33	0.0148	1	4
India	Dharkot, Uttarakhand, IN	IN7	2191.9	30.6558	78.2318	0.0778	0.25	0.0195	1	3
India	DPF Nanhani, Himachal Pradesh, IN	IN8	2675.8	31.7739	77.1002	0.1508	0.17	0.0256	1	2
India	Bahru, Himachal Pradesh, IN	IN9	1577.5	31.7318	76.7102	0.0562	0.25	0.0140	1	3
India	Chamba HP, IN	IN10	1731.4	32.4392	76.0730	0.0661	0.25	0.0165	1	3
India	Lad Bharol HP, IN	IN11	1577.5	31.9931	76.6059	0.0562	0.25	0.0140	1	3
India	Latti, Jammu and Kashmir, IN	IN12	1123.2	32.9708	75.4853	0.0757	0.17	0.0129	1	2
India	Nar Sher Ali Khan, Jammu and Kashmir, IN	IN13	1307.3	33.9424	73.9637	0.0478	0.17	0.0081	1	2
India	Bandipora, Jammu and Kashmir, IN	IN14	2763.9	34.4739	74.8261	0.0000	0	0.0000	0	0
India	Shimla, Himachal Pradesh, IN	IN15	2606.1	31.2983	77.8315	0.1135	0.08	0.0091	1	1

Region and Country	Location	Code	Elevation (m)	Latitude (°N)	Longitude (°E)	*Pst* MINF (A)	Fav-month (F)	Favourable index (A x F)	Risk score	Month
India	Rupal, Jammu and Kashmir, IN	IN16	3709.5	35.2421	74.9037	0.0051	0.08	0.0004	0	1
India	Sankoo, Jammu and Kashmir, IN	IN17	4017.7	34.2854	75.9144	0.0000	0	0.0000	0	0
India	Magh Batna, Jammu and Kashmir, IN	IN18	2745.7	33.2442	75.6507	0.0000	0	0.0000	0	0
India	Khawaspur, Punjab, IN	IN19	431.1	31.5018	75.9148	0.0337	0.08	0.0027	0	1
India	Dharm Manela, Himachal Pradesh, IN	IN20	4333.2	31.9359	78.3664	0.0403	0.08	0.0032	0	1
India	Jamaga, Karnataka, IN	IN21	498.9	15.1369	74.6675	0.0131	0.25	0.0033	0	3
India	Waynad, Kerala, IN	IN22	718.3	11.5457	76.2055	0.1863	0.33	0.0615	1	4
India	Thanjavur, Tamil Nadu, IN	IN23	51.8	10.8128	79.1059	0.0000	0	0.0000	0	0
India	Mendpal, Chhattisgarh, IN	IN24	517.4	19.2915	81.0395	0.0013	0.08	0.0001	0	1
India	Basnara, Chhattisgarh, IN	IN25	493.2	23.7059	82.7534	0.0026	0.08	0.0002	0	1
India	Adiwas, Rajasthan, IN	IN26	385.2	24.3481	73.3491	0.0000	0	0.0000	0	0
India	Khesari Gidha, Uttar Pradesh, IN	IN27	85.4	26.7662	83.9729	0.0000	0	0.0000	0	0
India	Nandhaur Range, Uttarakhand, IN	IN28	864.4	29.0764	79.7871	0.0116	0.33	0.0038	0	4
India	Deema Range, Uttarakhand, IN	IN29	891.5	29.5267	78.9082	0.0062	0.17	0.0011	0	2
India	Bahru, Himachal Pradesh, IN	IN30	1577.5	31.9438	76.5351	0.0562	0.25	0.0140	1	3
India	Bahl, Himachal Pradesh, IN	IN31	944.3	31.2889	76.6450	0.0318	0.08	0.0025	0	1
India	Golaghat, Assam, IN	IN32	369.8	26.5895	93.6299	0.0100	0.25	0.0025	0	3
India	Mokokchung, Nagaland, IN	IN33	668.8	26.2747	94.3769	0.0107	0.17	0.0018	0	2
India	Nagrasari, Rajasthan, IN	IN34	230.2	28.9995	74.7773	0.0000	0	0.0000	0	0
India	Jind, Haryana, IN	IN35	224.2	29.3449	76.3594	0.0000	0	0.0000	0	0
India	Thirpali Chhoti, Rajasthan, IN	IN36	312.1	28.4214	75.5244	0.0000	0	0.0000	0	0
India	Roranwala Urf Tarewali, Punjab	IN37	186.3	30.4875	74.2939	0.0000	0	0.0000	0	0
India	Gulbarga, Karnataka, IN	IN38	501.4	17.1996	77.2310	0.0000	0	0.0000	0	0
India	Poletipalle, Andhra Pradesh, IN	IN39	507.9	14.2814	77.6704	0.0000	0	0.0000	0	0
India	Marma, Maharashtra, IN	IN40	293.8	20.4435	80.4829	0.0000	0	0.0000	0	0
India	Kaimur, Bihar, IN	IN41	212.1	24.9422	83.6910	0.0000	0	0.0000	0	0
India	Karbi Anglog, Assam, IN	IN42	272.5	26.2452	93.3772	0.0061	0.17	0.0010	0	2
India	Champai, Mizoram, IN	IN43	955.7	23.4643	93.2453	0.0007	0.08	0.0001	0	1
India	Serchhip, Mizoram, IN	IN44	600.9	23.2525	92.9597	0.0004	0.08	0.0000	0	1

Region and Country	Location	Code	Elevation (m)	Latitude (°N)	Longitude (°E)	*Pst* MINF (A)	Fav-month (F)	Favourable index (A x F)	Risk score	Month
India	Baljata, J&K, IN	IN45	695.6	32.7513	74.9641	0.0372	0.17	0.0063	0	2
Mongolia	Unnamed, Lamaiin Huryee, MN	MN1	1646.8	43.7402	103.2539	0.0000	0	0.0000	0	0
Mongolia	Unnamed, Khurmen, MN	MN2	1210.1	42.2619	103.4626	0.0000	0	0.0000	0	0
Mongolia	Unnamed, Shinejinst, MN	MN3	1208.9	43.8433	98.9033	0.0020	0.08	0.0002	0	1
Mongolia	Unnamed, Munkhkhairkhan, MN	MN4	2520.2	46.9285	91.6304	0.0011	0.08	0.0001	0	1
Mongolia	Unnamed, Tsagaankhairkhan, MN	MN5	1225.3	48.8727	93.9155	0.0103	0.17	0.0017	0	2
Mongolia	Unnamed, Orkhon, MN	MN6	1337.4	48.6699	103.9790	0.0095	0.08	0.0008	0	1
Mongolia	Unnamed, Dashbalbar, MN	MN7	820.7	49.2470	114.4380	0.0033	0.08	0.0003	0	1
Mongolia	Unnamed, Matad, MN	MN8	736.1	46.8985	116.0639	0.0000	0	0.0000	0	0
Mongolia	Unnamed, Luus, MN	MN9	1307.8	45.2525	105.7805	0.0000	0	0.0000	0	0
Mongolia	Unnamed, Khuvsgul, MN	MN10	950.7	43.6210	109.9116	0.0000	0	0.0000	0	0
Myanmar	Sagaing Region 1, Mym	MYN1	344.5	24.6805	95.8436	0.0000	0	0.0000	0	0
Myanmar	Kyaukme, Mym	MYN2	950.9	22.7494	97.5575	0.0000	0	0.0000	0	0
Myanmar	Mindat, Mym	MYN3	932.5	21.4439	93.5749	0.0000	0	0.0000	0	0
Myanmar	Kyaukpyu, Mym	MYN4	315.1	19.7264	94.1682	0.0000	0	0.0000	0	0
Myanmar	Rakhine, Mym	MYN5	42.4	17.4041	94.7615	0.0000	0	0.0000	0	0
Myanmar	Putao 1, Mym	MYN6	924.5	26.5994	97.4421	0.0121	0.25	0.0030	0	3
Myanmar	Bhamo, Mym	MYN7	606.2	24.4182	97.0027	0.0000	0	0.0000	0	0
Myanmar	Sagaing Region 2, Mym	MYN8	299.1	21.9950	94.8933	0.0000	0	0.0000	0	0
Nepal	Guthichaur, NP	NP1	3284.5	29.1382	82.3183	0.1301	0.17	0.0221	1	2
Nepal	Namarjung, NP	NP2	2339.8	28.4305	84.2354	0.2502	0.25	0.0626	1	3
Nepal	Maipokhari, NP	NP3	2076.8	27.0210	87.8950	0.1539	0.17	0.0262	1	2
Nepal	Seti, NP	NP4	1877.7	29.4053	81.0641	0.0815	0.17	0.0138	1	2
Nepal	Phopli 1, NP	NP5	1618.9	28.2086	82.8523	0.1004	0.25	0.0251	1	3
Nepal	Phopli 2, NP	NP6	1116.5	29.0152	80.3625	0.0450	0.33	0.0148	1	4
Nepal	Kailali, NP	NP7	601.5	28.9239	80.5225	0.0030	0.08	0.0002	0	1
Nepal	Bheri, NP	NP8	630.9	28.4435	81.5676	0.0000	0	0.0000	0	0
Nepal	Chitwan, NP	NP9	1142.1	27.7651	84.5600	0.0732	0.33	0.0242	1	4
Nepal	Firfire, NP	NP10	2136.2	28.0855	83.9832	0.2038	0.25	0.0510	1	3

Region and Country	Location	Code	Elevation (m)	Latitude (°N)	Longitude (°E)	*Pst* MINF (A)	Fav-month (F)	Favourable index (A x F)	Risk score	Month
Nepal	Huktumba, NP	NP11	706.1	27.8963	83.3130	0.0126	0.08	0.0010	0	1
Nepal	Khalanga, NP	NP12	630.9	28.4214	81.9025	0.0005	0.08	0.0000	0	1
Nepal	Tilepata, NP	NP13	2451.5	29.0476	81.5010	0.1199	0.17	0.0204	1	2
Nepal	Khalanga NP	NP14	630.9	28.4214	81.9075	0.0000	0	0.0000	0	0
Nepal	Malatikot NP	NP15	2451.5	29.0476	81.5010	0.0937	0.17	0.0159	1	2
Pakistan	Baricot, Khyber Pakhtunkhwa, PK	PK1	1457.6	34.7064	72.2834	0.0992	0.17	0.0169	1	2
Pakistan	Abbottabad, Khyber Pakhtunkhwa, PK	PK2	1418.5	34.2173	73.2722	0.0722	0.17	0.0123	1	2
Pakistan	Jand, Punjab, PK	PK3	460.3	33.6339	72.1955	0.0296	0.17	0.0050	0	2
Pakistan	Darsmand, Khyber Pakhtunkhwa, PK	PK4	896.8	33.4140	70.6575	0.0546	0.17	0.0093	1	2
Pakistan	Wana 1, Waziristan, PK	PK5	754.7	31.9531	70.1081	0.0118	0.17	0.0020	0	2
Pakistan	Qila Saifullah, Balochistan, PK	PK6	1916.1	30.7145	68.3723	0.0036	0.08	0.0003	0	1
Pakistan	Wana 2, Waziristan, PK	PK7	1346.1	32.3809	69.6247	0.0200	0.17	0.0034	0	2
Pakistan	Awaran 1, Balochistan, PK	PK8	347.2	25.7811	64.9006	0.0000	0	0.0000	0	0
Pakistan	Khuzdar, Balochistan, PK	PK9	1419.9	28.0730	66.6804	0.0000	0	0.0000	0	0
Pakistan	Sangan, Balochistan, PK	PK10	913.3	29.9369	67.7351	0.0029	0.08	0.0002	0	1
Pakistan	Kalkot, Khyber Pakhtunkhwa, PK	PK11	2661.5	35.3721	72.1736	0.0065	0.08	0.0005	0	1
Pakistan	Awaran 2, Balochistan, PK	PK12	714.4	26.0775	64.9919	0.0000	0	0.0000	0	0
Pakistan	Mastuj, Khyber Pakhtunkhwa, PK	PK13	3641.5	35.8677	72.2889	0.0000	0	0.0000	0	0
Pakistan	Hunza Nagar, PK	PK14	4536.9	36.2053	75.1893	0.0000	0	0.0000	0	0
Pakistan	Alai, Khyber Pakhtunkhwa, PK	PK15	2219.3	34.8823	73.1019	0.0000	0	0.0000	0	0
Pakistan	Bara, Khyber Agency, PK	PK16	1322.9	33.7755	70.8168	0.0712	0.17	0.0121	1	2
	**Proportion of sites with infection index**	**0.756**								
**Central Asia (195)**										
Afghanistan	Khas Uruzgan, AFG	AFG1	2155.9	32.9036	66.4881	0.0000	0	0.0000	0	0
Afghanistan	Berana, Panjab, AFG	AFG2	3161.5	34.4531	67.2187	0.0000	0	0.0000	0	0
Afghanistan	Kohistan, AFG	AFG3	2240.4	35.3989	64.8347	0.0000	0	0.0000	0	0
Afghanistan	Balkh, AFG	AFG4	1416.8	36.3470	67.0375	0.0057	0.25	0.0014	0	3
Afghanistan	Warsaj, AFG	AFG5	3346.1	36.2939	70.2455	0.0051	0.08	0.0004	0	1

Region and Country	Location	Code	Elevation (m)	Latitude (°N)	Longitude (°E)	*Pst* MINF (A)	Fav-month (F)	Favourable index (A x F)	Risk score	Month
Afghanistan	Alingar 1, AFG	AFG6	1771.3	34.8643	70.5970	0.0382	0.33	0.0126	1	4
Afghanistan	Pur Chaman, AFG	AFG7	1906.7	32.8298	64.0272	0.0015	0.08	0.0001	0	1
Afghanistan	Alingar 2, AFG	AFG8	2061.5	34.7245	70.3498	0.0000	0	0.0000	0	0
Afghanistan	Sabari, AFG	AFG9	1903.8	33.4690	69.9983	0.0082	0.17	0.0014	0	2
Afghanistan	Bargi Matal, AFG	AFG10	3682.4	35.6582	71.0530	0.0000	0	0.0000	0	0
Afghanistan	Panjwai, AFG	AFG11	1083.6	31.3171	65.3181	0.0000	0	0.0000	0	0
Afghanistan	Taywara, AFG	AFG12	2370.5	33.3957	64.1535	0.0000	0	0.0000	0	0
Afghanistan	Sayyad, AFG	AFG13	1103.6	36.0855	66.0432	0.0064	0.17	0.0011	0	2
Afghanistan	Bangi, AFG	AFG14	1641.3	36.5469	69.5368	0.0156	0.25	0.0039	0	3
Afghanistan	Zinda Jan, AFG	AFG15	1012.4	34.6703	61.8684	0.0006	0.08	0.0001	0	1
Afghanistan	Khamyab, AFG	AFG16	302.8	37.3361	65.8894	0.0098	0.25	0.0025	0	3
Afghanistan	Baghran, AFG	AFG17	2147.4	33.2121	65.0544	0.0000	0	0.0000	0	0
Afghanistan	Chihil Baghtoe Pashi, AFG	AFG18	2885.7	33.0833	67.2077	0.0000	0	0.0000	0	0
Afghanistan	Saghar, AFG	AFG19	2355.2	33.5469	63.5054	0.0000	0	0.0000	0	0
Afghanistan	Khidir, AFG	AFG20	2580.1	33.7298	65.4939	0.0000	0	0.0000	0	0
Azerbaijan	Alekseyevka, Khasmaz, AZ	AZ1	70.9	41.5036	48.6896	0.6328	0.83	0.5252	3	10
Azerbaijan	Derk, Quba, AZ	AZ2	1291.3	41.0412	48.3820	0.0149	0.17	0.0025	0	2
Azerbaijan	Qaradagh, Baku, AZ	AZ3	161.1	40.3247	49.4916	0.1921	0.5	0.0961	1	6
Azerbaijan	Burkandul, Lerik, AZ	AZ4	461.7	38.8431	48.5468	0.3390	0.67	0.2271	2	8
Azerbaijan	Hasanabad, Neftchala, AZ	AZ5	-3.6	39.3546	49.1730	0.9570	0.83	0.7943	4	10
Azerbaijan	Kurdchu, Hajiqabul, AZ	AZ6	1.2	39.9215	49.2279	0.3589	0.67	0.2405	2	8
Azerbaijan	Malikumudlu, Ujar, AZ	AZ7	90.9	40.3331	47.7668	0.0511	0.17	0.0087	1	2
Azerbaijan	Nagorno-Karabakh, AZ	AZ8	790.1	40.0730	46.5802	0.0220	0.25	0.0055	0	3
Azerbaijan	Alxanli, Fizuli, AZ	AZ9	409.2	39.6682	47.3493	0.0457	0.33	0.0151	1	4
Azerbaijan	Sarica, Shaki, AZ	AZ10	426.9	40.9168	47.3273	0.0602	0.33	0.0199	1	4
Azerbaijan	Qinli, Agstafa, AZ	AZ11	567.3	41.1157	45.5695	0.0597	0.42	0.0251	1	5
Azerbaijan	Damchili, Goygol, AZ	AZ12	823.8	40.6172	46.2177	0.0314	0.33	0.0104	1	4
Azerbaijan	Istisu, Nagorno-Karabakh, AZ	AZ13	2067.1	39.8793	45.9320	0.0000	0	0.0000	0	0
Azerbaijan	Dizavar, Khizi, AZ	AZ14	289.5	40.8575	49.1953	0.2393	0.58	0.1388	2	7

Region and Country	Location	Code	Elevation (m)	Latitude (°N)	Longitude (°E)	*Pst* MINF (A)	Fav-month (F)	Favourable index (A x F)	Risk score	Month
Azerbaijan	Khinaliq, Qusar, AZ	AZ15	1291.3	41.2291	48.0146	0.0149	0.17	0.0025	0	2
Azerbaijan	Solguja, Oghuz, AZ	AZ16	1526.9	41.0732	47.6462	0.0149	0.17	0.0025	0	2
Iran	Ahar, East Azerbaijan, IRN	IRN1	1361.2	38.6521	47.0368	0.0000	0	0.0000	0	0
Iran	Tang-e-Panj, Khuzestan, IRN	IRN2	989.6	32.9543	48.6625	0.0134	0.17	0.0023	0	2
Iran	Aghowz Darban, Mazandaran, IRN	IRN3	1997.9	36.4575	51.2117	0.0231	0.25	0.0058	0	3
Iran	Hakan, Fars, IRN	IRN4	1420.5	28.6530	53.2771	0.0000	0	0.0000	0	0
Iran	Far-Ashian, North Khorasan, IRN	IRN5	1180.9	36.7750	56.8367	0.0000	0	0.0000	0	0
Iran	Ferdows, South Khorasan, IRN	IRN6	1186.8	33.9807	57.9813	0.0800	0.5	0.0400	1	6
Iran	Gale Koor, Lorestan, IRN	IRN7	1555.3	32.8720	49.2840	0.0000	0	0.0000	0	0
Iran	Tabriz, East Azerbaijan, IRN	IRN8	1562.5	38.1117	45.9190	0.0211	0.17	0.0036	0	2
Iran	Basak, Gilan, IRN	IRN9	1263.7	37.8692	48.7754	0.1121	0.5	0.0560	1	6
Iran	Arab Lang, Kurdistan, IRN	IRN10	1674.5	36.0789	46.2266	0.0037	0.08	0.0003	0	1
Iran	West Azerbaijan, IRN	IRN11	1872.8	38.0079	44.6446	0.0011	0.08	0.0001	0	1
Iran	Bilesavar, Ardabil, IRN	IRN12	587.1	39.2778	47.8965	0.0339	0.33	0.0112	1	4
Iran	Kharfegil, Gilan, IRN	IRN13	1263.7	37.6956	48.9073	0.1121	0.5	0.0560	1	6
Iran	Charoymaq, East Azerbaijan, IRN	IRN14	1767.6	37.2771	47.1495	0.0000	0	0.0000	0	0
Iran	Kafteroud, Gilan, IRN	IRN15	451.9	37.3470	49.5226	0.4981	0.75	0.3736	3	9
Iran	Gadel, Zanjan, IRN	IRN16	1773.4	36.6098	47.9405	0.0000	0	0.0000	0	0
Iran	Roudbar Alamout, Qazvin, IRN	IRN17	1247.7	36.5745	50.0498	0.0748	0.5	0.0374	1	6
Iran	Tamisan, Tehran, IRN	IRN18	1912.7	35.5801	51.9834	0.0078	0.17	0.0013	0	2
Iran	Qarehtappeh, Mazandaran, IRN	IRN19	228.1	36.7508	53.4336	0.3610	0.75	0.2708	2	9
Iran	Narlidaq, Golestan, IRN	IRN20	203.9	37.6956	55.2793	0.0927	0.5	0.0463	1	6
Iran	Shirvan, North Khorasan, IRN	IRN21	1261.9	37.5215	57.8721	0.0081	0.17	0.0014	0	2
Iran	Mozduran, Razavi Khorasan, IRN	IRN22	682.3	36.0789	60.5528	0.0229	0.17	0.0039	0	2
Iran	Shahrud, Semnan, IRN	IRN23	996.5	35.9723	56.5538	0.0000	0	0.0000	0	0
Iran	Abadan, Khuzestan, IRN	IRN24	2.5	30.3478	48.6436	0.0000	0	0.0000	0	0
Iran	Anjiru, Bushehr, IRN	IRN25	113.3	29.4334	50.7530	0.1163	0.33	0.0384	1	4
Iran	Abdan, Bushehr, IRN	IRN26	125.1	27.9687	51.7198	0.0324	0.25	0.0081	1	3
Iran	Garisheh, Hormozgan, IRN	IRN27	217.3	26.9157	53.7413	0.0000	0	0.0000	0	0

Region and Country	Location	Code	Elevation (m)	Latitude (°N)	Longitude (°E)	*Pst* MINF (A)	Fav-month (F)	Favourable index (A x F)	Risk score	Month
Iran	Tifakan, Hormozgan, IRN	IRN28	795.5	27.6967	56.2901	0.0000	0	0.0000	0	0
Iran	Kasi, Sistan and Balochistan, IRN	IRN29	159.1	25.7341	59.1026	0.0000	0	0.0000	0	0
Iran	Khash, Sistan and Balochistan, IRN	IRN30	1293.8	28.0851	61.7393	0.0000	0	0.0000	0	0
Iran	Zarand, Kerman, IRN	IRN31	1788.7	30.7263	56.3780	0.0000	0	0.0000	0	0
Iran	Reza Abad, Fars, IRN	IRN32	2109.6	30.4236	52.6426	0.0029	0.08	0.0002	0	1
Iran	Sirusabad, Yazd, IRN	IRN33	1461.3	31.6660	55.8506	0.0000	0	0.0000	0	0
Iran	Kashan County, Isfahan, IRN	IRN34	1863.2	33.7001	51.2364	0.0000	0	0.0000	0	0
Iran	Jera, Hamadan, IRN	IRN35	1869.8	34.4282	48.2920	0.0064	0.08	0.0005	0	1
Iran	Cheshme, Markazi, IRN	IRN36	1774.3	34.2831	50.1817	0.0000	0	0.0000	0	0
Iran	Mahdishahr, Semnan, IRN	IRN37	1526.6	35.7586	53.5215	0.0011	0.08	0.0001	0	1
Iran	Shahrud 2, Semnan, IRN	IRN38	812.5	35.0781	55.5870	0.0024	0.08	0.0002	0	1
Iran	Dehrud, Fars, IRN	IRN39	1336.8	28.5759	52.5225	0.0000	0	0.0000	0	0
Iran	Urmia, West Azerbaijan, IRN	IRN40	1888.9	37.7890	44.7441	0.0132	0.17	0.0022	0	2
Iran	Chalus, Mazandaran, IRN	IRN41	819.2	36.5825	51.4475	0.6182	0.83	0.5131	3	10
Iran	Pourva, Mazandaran, IRN	IRN42	228.1	36.5296	53.4800	0.3610	0.75	0.2708	2	9
Iran	Lulak-E Lebur, Mazandaran, IRN	IRN43	1110.9	36.2820	53.0735	0.0498	0.42	0.0209	1	5
Iran	Pust Kola, Mazandaran, IRN	IRN44	1168.4	36.2909	52.6560	0.0800	0.5	0.0400	1	6
Iran	Manuchehrkela, Mazandaran, IRN	IRN45	508.3	36.5031	51.8760	0.9217	0.83	0.7650	4	10
Iran	Shalma Shaft, Gilan, IRN	IRN46	692.2	37.1013	49.2942	0.2967	0.75	0.2225	2	9
Iran	Askabon, Gilan, IRN	IRN47	1202.6	36.6443	49.7446	0.0459	0.42	0.0193	1	5
Iran	GolRoudbar, Gilan, IRN	IRN48	451.9	37.1451	49.9534	0.4981	0.75	0.3736	3	9
Iran	Harandan, Gilan, IRN	IRN49	925.4	38.1368	48.8438	0.2073	0.67	0.1389	2	8
Iran	Rudakanar, Masally, AZ	IRN50	461.7	38.8847	48.6790	0.3390	0.67	0.2271	2	8
Iran	Shaft, Gilan, IRN	IRN51	692.2	37.0421	49.4178	0.2967	0.75	0.2225	2	9
Iran	Shiveh Berow, West Azerbaijan, IRN	IRN52	1646.2	37.1648	45.0562	0.0267	0.25	0.0067	0	3
Iran	Akhond Mahalleh, Mazandaran, IRN	IRN53	1152.9	36.7786	50.8131	0.2804	0.83	0.2328	2	10
Iran	Chanasak, Qazvin, IRN	IRN54	1617.6	36.2842	50.3516	0.0037	0.08	0.0003	0	1
Iran	Kuchek Khortum, Golestan, IRN	IRN55	322.6	37.1122	54.9439	0.0921	0.5	0.0460	1	6
Iran	Viva, Golestan, IRN	IRN56	556.3	36.6376	53.9551	0.2426	0.67	0.1626	2	8

Region and Country	Location	Code	Elevation (m)	Latitude (°N)	Longitude (°E)	*Pst* MINF (A)	Fav-month (F)	Favourable index (A x F)	Risk score	Month
Iran	Paeen Ahmad, Mazandaran, IRN	IRN57	171.2	36.5318	52.5269	0.5580	0.75	0.4185	3	9
Iran	Zirab, Mazandaran, IRN	IRN58	1168.4	36.2311	52.9444	0.0000	0	0.0000	0	0
Kazakhstan	Aksu, Karakiya, KZ	KZ1	82.1	43.2940	52.2334	0.0010	0.08	0.0001	0	1
Kazakhstan	Karakum, Tupkaragan, KZ	KZ2	21.9	44.4032	50.6513	0.2672	0.58	0.1550	2	7
Kazakhstan	Unnamed 2, Karakiya, KZ	KZ3	184.9	43.8670	54.4306	0.0000	0	0.0000	0	0
Kazakhstan	Unnamed, Karakum, Tupkaragan, KZ	KZ4	-24.1	45.2138	51.7500	0.0802	0.5	0.0401	1	6
Kazakhstan	Unnamed, Isatay, KZ	KZ5	-21.7	46.4083	53.5517	0.0000	0	0.0000	0	0
Kazakhstan	Kulandy, Aral, KZ	KZ6	96.1	46.1350	59.3965	0.0276	0.25	0.0069	0	3
Kazakhstan	Bis-Bulyuk, Makat, KZ	KZ7	-22	47.3098	53.2441	0.0064	0.08	0.0005	0	1
Kazakhstan	Kimer-Sayek-Kul', Syrym, KZ	KZ8	20.8	50.4022	51.8818	0.0432	0.33	0.0143	1	4
Kazakhstan	Akhol, Ayteke Bi, KZ	KZ9	264.6	50.0085	60.5830	0.0420	0.17	0.0071	0	2
Kazakhstan	Aiyrtau, Airtau, KZ	KZ10	298.8	53.1211	68.3613	0.1355	0.42	0.0569	1	5
Kazakhstan	L'vovka, Kashyr, KZ	KZ11	102.5	53.2527	76.4472	0.0811	0.33	0.0268	1	4
Kazakhstan	Sartymbet, Ulan, KZ	KZ12	469.3	49.6684	81.9844	0.0349	0.33	0.0115	1	4
Kazakhstan	Unnamed, Bukhar-Zhyrau, KZ	KZ13	729.3	49.4117	73.8105	0.0268	0.25	0.0067	0	3
Kazakhstan	Unnamed, Nura, KZ	KZ14	451.8	49.9237	67.5263	0.0490	0.25	0.0122	1	3
Kazakhstan	Unnamed, South Kazakhstan, KZ	KZ15	217.2	42.2944	67.7021	0.0098	0.08	0.0008	0	1
Kazakhstan	Unnamed, Bayganin, KZ	KZ16	346.2	46.1958	57.1289	0.0587	0.42	0.0247	1	5
Kazakhstan	Goloschchekin, Atyrau, KZ	KZ17	-23.7	46.5022	49.2310	0.0185	0.25	0.0046	0	3
Kazakhstan	Unnamed, Atyrau, KZ	KZ18	-18.1	47.7105	52.5025	0.0250	0.17	0.0043	0	2
Kyrgyzstan	Issyk-Kul, KGN	KGN1	2135.8	42.6611	76.4720	0.0276	0.17	0.0047	0	2
Kyrgyzstan	Temir-Kanat, Tong, KGN	KGN2	2466.9	42.0359	76.9224	0.0600	0.25	0.0150	1	3
Kyrgyzstan	Ak-Su 1, KGN	KGN3	3217.1	42.4020	78.9988	0.0000	0	0.0000	0	0
Kyrgyzstan	Naryn, At-Bashi, KGN	KGN4	3383.3	40.9496	75.7798	0.0000	0	0.0000	0	0
Kyrgyzstan	Lyangar, Kadamzhai, KGN	KGN5	2798.9	39.7440	71.9456	0.0000	0	0.0000	0	0
Kyrgyzstan	Uzak, Aravan, KGN	KGN6	2162.3	40.3662	72.7586	0.0417	0.33	0.0138	1	4
Kyrgyzstan	Terek, Ak-Talaa, KGN	KGN7	2676.5	41.1981	74.9119	0.0012	0.08	0.0001	0	1
Kyrgyzstan	Karabashat 1, Aksy, KGN	KGN8	2140.5	41.7250	71.7478	0.0000	0	0.0000	0	0
Kyrgyzstan	Ak-Su 2, KGN	KGN9	3383.9	42.0685	79.6580	0.0036	0.17	0.0006	0	2

						Pst				
Region and Country	Location	Code	Elevation (m)	Latitude (°N)	Longitude (°E)	MINF (A)	Fav-month (F)	Favourable index (A x F)	Risk score	Month
Kyrgyzstan	Konduk, Kara-Kulja, KGN	KGN10	2845.5	40.5648	74.0303	0.0000	0	0.0000	0	0
Kyrgyzstan	Karabashat 2, Aksy, KGN	KGN11	2140.5	41.7230	71.7231	0.0000	0	0.0000	0	0
Kyrgyzstan	Panfilov, KGN	KGN12	2698.5	42.2944	74.6675	0.0036	0.17	0.0006	0	2
Kyrgyzstan	Chot-Nura, Naryn, KGN	KGN13	2861.1	41.6738	76.3374	0.0000	0	0.0000	0	0
Kyrgyzstan	Issyk-kul region, KGN	KGN14	2672.9	42.2972	79.4640	0.0000	0	0.0000	0	0
Russia	Rebrikhinsky, Altai Krai, RS	RS1	197.4	53.2790	82.3359	0.0839	0.33	0.0277	1	4
Russia	Miguty, Krasnodor Krai, RS	RS2	32.9	46.1350	39.0937	0.0772	0.33	0.0255	1	4
Russia	Indyuk, Krasnodor Krai, RS	RS3	312.4	44.2617	39.2036	0.5044	0.75	0.3783	3	9
Russia	Rassvet, Volgograd Oblast, RS	RS4	78.7	47.5180	43.3125	0.0654	0.33	0.0216	1	4
Russia	Tyurinsky, Volgograd Oblast, RS	RS5	118.2	50.0508	42.6094	0.0518	0.33	0.0171	1	4
Russia	Sadovyy, Volgograd Oblast, RS	RS6	19.8	49.4689	46.6094	0.0590	0.25	0.0147	1	3
Russia	Grabovskiy, Rostov Oblast, RS	RS7	94.9	46.7858	43.2466	0.0458	0.33	0.0151	1	4
Russia	Nogaysky, Dagetsan Republic, RS	RS8	-12.1	44.5286	46.1689	0.0188	0.08	0.0015	0	1
Russia	Verkh Karanay, Dagestan Reoublic, RS	RS9	1152.5	42.8607	46.8281	0.0547	0.33	0.0181	1	4
Russia	Novourupskiy, Krasnodar Krai, RS	RS10	746.8	44.3718	41.5766	0.0888	0.5	0.0444	1	6
Russia	Indyuk, Krasnodor Krai, RS	RS11	312.4	44.2303	39.3135	0.5044	0.75	0.3783	3	9
Russia	Kiziltashskiy Liman, Krasnodar Krai, RS	RS12	24.5	45.1518	37.0283	0.7599	0.67	0.5091	3	8
Russia	Oktyabr'skiy, Krasnodar Krai, RS	RS13	7.3	46.4840	38.2368	0.3181	0.67	0.2131	2	8
Russia	Sadovyy, Volgograd Oblast, RS	RS14	112.5	48.0348	40.7197	0.0345	0.25	0.0086	1	3
Russia	Grabovskiy, Rostov Oblast, RS	RS15	701.6	54.7062	88.2905	0.2580	0.33	0.0851	1	4
Russia	Nogaysky, Dagetsan Republic, RS	RS16	1393.6	55.9067	114.4819	0.0495	0.33	0.0163	1	4
Russia	Verkh Karanay, Dagestan Reoublic, RS	RS17	1157.1	55.7092	132.4116	0.1149	0.25	0.0287	1	3
Russia	Novourupskiy, Krasnodar Krai, RS	RS18	615.7	45.9901	135.5757	0.0631	0.33	0.0208	1	4
Russia	Goytkh, Krasnodar Krai, RS	RS19	393.9	55.7092	59.2866	0.2473	0.5	0.1236	2	6
Russia	Volodarsky, Astrakhan Oblast, RS	RS20	-26.7	46.2730	48.7559	0.0480	0.42	0.0201	1	5
Russia	Kalmykia, RS	RS21	-6.9	46.6061	46.3828	0.0179	0.33	0.0059	0	4
Russia	Grushevo, Astrakhan Oblast, RS	RS22	-25.3	46.0523	48.4840	0.0094	0.25	0.0024	0	3
Russia	Khivsky, Dagestan, RS	RS23	699.9	41.7734	48.0088	0.1080	0.5	0.0540	1	6
Tajikistan	Ardzhanak, Hisor, TJ	TJ1	2031.3	38.7207	68.5810	0.0043	0.08	0.0003	0	1

Region and Country	Location	Code	Elevation (m)	Latitude (°N)	Longitude (°E)	*Pst* MINF (A)	Fav-month (F)	Favourable index (A x F)	Risk score	Month
Tajikistan	Kaltashur, TJ	TJ2	798.6	37.8931	69.0534	0.0423	0.25	0.0106	1	3
Tajikistan	Arabkhana, Shahrtuz, TJ	TJ3	527.8	37.1875	68.0976	0.0206	0.33	0.0068	0	4
Tajikistan	Unnamed, Gorno-Badakhshan, TJ	TJ4	4356.1	37.9624	74.4148	0.0000	0	0.0000	0	0
Tajikistan	Khamirabot, Ghafurov, TJ	TJ5	1040.6	40.3976	69.6028	0.0267	0.33	0.0088	1	4
Tajikistan	Karakendzha, Jirgatol, TJ	TJ6	3683.9	39.2331	71.6023	0.0000	0	0.0000	0	0
Tajikistan	Shahrinav, Shakhrinaw, TJ	TJ7	2177.9	38.5662	68.3284	0.0000	0	0.0000	0	0
Tajikistan	Medynved, Roshtqala, TJ	TJ8	3993.2	37.1437	71.9538	0.0000	0	0.0000	0	0
Tajikistan	Vose, TJ	TJ9	1258.5	37.8063	69.6028	0.0231	0.25	0.0058	0	3
Tajikistan	Shurpast, Varzov, TJ	TJ10	2674.8	39.0115	68.8227	0.0000	0	0.0000	0	0
Tajikistan	Qurgonteppa, Sarband, TJ	TJ11	627.2	37.7890	68.9546	0.0372	0.33	0.0123	1	4
Tajikistan	Arzing, Tavildara, TJ	TJ12	3372.1	38.8748	71.2837	0.0000	0	0.0000	0	0
Tajikistan	Barchidev, Rushon, TJ	TJ13	4442.3	38.3253	72.5141	0.0000	0	0.0000	0	0
Turkmenistan	Karagez, Balkan, TKM	TKM1	194.8	38.9723	55.3614	0.0565	0.25	0.0141	1	3
Turkmenistan	Syul'men, Balkan, TKM	TKM2	37.1	40.3474	53.3869	0.0502	0.25	0.0125	1	3
Turkmenistan	Aydin, Balkan, TKM	TKM3	100.3	39.4455	54.9031	0.0402	0.25	0.0101	1	3
Turkmenistan	Ekerem, Balkan, TKM	TKM4	-22.5	38.0836	53.9802	0.4088	0.67	0.2739	2	8
Turkmenistan	Bekibent, Balkan, TKM	TKM5	194.8	38.5147	55.2326	0.0565	0.25	0.0141	1	3
Turkmenistan	Kabyl, Balkan, TKM	TKM6	37.1	40.3641	53.0793	0.0502	0.25	0.0125	1	3
Turkmenistan	Unnamed 1, Balkan, TKM	TKM7	124.2	40.6315	54.7053	0.0000	0	0.0000	0	0
Turkmenistan	Unnamed 2, Balkan, TKM	TKM8	144.2	41.7803	54.1670	0.0000	0	0.0000	0	0
Turkmenistan	Kaplankyr Reserve1, Dashoguz, TKM	TKM9	78.6	40.7731	57.8254	0.0000	0	0.0000	0	0
Turkmenistan	Kaplankyr Reserve 2, Dashoguz, TKM	TKM10	42.3	41.9603	57.8364	0.0060	0.08	0.0005	0	1
Turkmenistan	Shakhnamy, Dasoguz, TKM	TKM11	84.4	42.0338	59.7700	0.0014	0.08	0.0001	0	1
Turkmenistan	Unnamed 1, Mary, TKM	TKM12	611.1	35.7385	62.3738	0.0118	0.17	0.0020	0	2
Turkmenistan	Dervezekem, Mary, TKM	TKM13	712.3	36.3691	64.1975	0.0158	0.17	0.0027	0	2
Turkmenistan	Batash, Lebap, TKM	TKM14	394.8	37.9451	65.6257	0.0091	0.33	0.0030	0	4
Turkmenistan	Unnamed 2, Mary, TKM	TKM15	191.8	38.4717	62.3078	0.0107	0.25	0.0027	0	3
Turkmenistan	Unnamed , Ahal, TKM	TKM16	137.3	39.3607	60.1655	0.0204	0.25	0.0051	0	3
Turkmenistan	Serbar, Balkan, TKM	TKM17	359.5	38.9603	56.1116	0.0394	0.17	0.0067	0	2

Region and Country	Location	Code	Elevation (m)	Latitude (°N)	Longitude (°E)	*Pst* MINF (A)	Fav-month (F)	Favourable index (A x F)	Risk score	Month
Turkmenistan	Unnamed 3, Balkan, TKM	TKM18	151.7	40.0537	55.8589	0.0000	0	0.0000	0	0
Turkmenistan	Unnamed, Dashoguz, TKM	TKM19	68.5	41.0222	57.2541	0.0000	0	0.0000	0	0
Turkmenistan	Unnamed 4, Balkan, TKM	TKM20	87.4	39.9527	56.4302	0.0266	0.17	0.0045	0	2
Uzbekistan	Shahrisabz, Yakkabog, UZ	UZ1	1266.3	38.9944	66.8232	0.0127	0.17	0.0022	0	2
Uzbekistan	Unnamed, Tamdy, UZ	UZ2	268.1	42.8446	64.4941	0.0121	0.08	0.0010	0	1
Uzbekistan	Unnamed 1, Muynak, UZ	UZ3	49.6	43.9936	60.1435	0.0685	0.33	0.0226	1	4
Uzbekistan	Unnamed 1, Kungrad, UZ	UZ4	156.5	44.8099	57.9023	0.0048	0.17	0.0008	0	2
Uzbekistan	Unnamed 2, Kungrad, UZ	UZ5	90.5	41.9685	56.6719	0.0062	0.08	0.0005	0	1
Uzbekistan	Vozrozhdeniya, UZ	UZ6	34.7	44.9345	59.1328	0.4814	0.67	0.3225	3	8
Uzbekistan	Unnamed 3, Kungrad, UZ	UZ7	126.4	45.0588	57.9023	0.0030	0.08	0.0002	0	1
Uzbekistan	Unnamed 4, Kungrad, UZ	UZ8	108.2	45.2757	58.0781	0.0560	0.33	0.0185	1	4
Uzbekistan	Unnamed 1, Karakiya, KZ	UZ9	132.7	42.4891	54.0791	0.0000	0	0.0000	0	0
Uzbekistan	Unnamed 2, Muynak, UZ	UZ10	37.1	44.0252	59.0010	0.4651	0.67	0.3116	3	8
Uzbekistan	Unnamed 3, Muynak, UZ	UZ11	58.8	43.5175	58.7373	0.0459	0.33	0.0152	1	4
Uzbekistan	Kungrad, UZ	UZ12	150.2	44.1400	56.4082	0.0200	0	0.0000	0	0
Uzbekistan	Ahangaran, UZ	UZ13	1168.2	41.1319	69.9681	0.1250	0.33	0.0413	1	4
	**Proportion of sites with infection index**	**0.728**								
**Northwest Asia (59)**										
Armenia	Lernahovit, Gegharkunik, ARM	ARM1	2029.7	40.0646	45.2619	0.0233	0.17	0.0040	0	2
Armenia	Norekart, Gegharkunik, ARM	ARM2	1988.9	40.2493	45.6903	0.0110	0.17	0.0019	0	2
Armenia	Gandzakar, Tavush, ARM	ARM3	1475.6	40.8254	45.2179	0.0395	0.17	0.0067	0	2
Armenia	Krasar, Shirak, ARM	ARM4	2070.9	41.0080	43.8007	0.0104	0.17	0.0018	0	2
Armenia	Armavir, Armavir, ARM	ARM5	1559.4	40.1570	43.9875	0.0096	0.17	0.0016	0	2
Armenia	Semyonovka, Gegharkunik, ARM	ARM6	1737.4	40.6256	44.8773	0.0275	0.17	0.0047	0	2
Armenia	Daranak, Gegharkunik, ARM	ARM7	1988.9	40.3749	45.5915	0.0110	0.17	0.0019	0	2
Armenia	Artsvanist, Gegharkunik, ARM	ARM8	1988.9	40.1318	45.5255	0.0110	0.17	0.0019	0	2
Armenia	Tsovazard, Gegharkunik, ARM	ARM9	2029.8	40.4502	45.0312	0.0233	0.17	0.0040	0	2
Armenia	Dilijan, Bovakar Ridge, ARM	ARM10	1737.4	40.8479	44.8725	0.0275	0.17	0.0047	0	2

Region and Country	Location	Code	Elevation (m)	Latitude (°N)	Longitude (°E)	*Pst* MINF (A)	Fav-month (F)	Favourable index (A x F)	Risk score	Month
Georgia	Likhni, GRG	GRG1	996.6	43.1602	40.6256	0.5499	0.83	0.4564	3	10
Georgia	Lukhi, Samegrelo-Zemo Svaneti, GRG	GRG2	1259.3	42.9837	42.1308	0.1193	0.58	0.0692	1	7
Georgia	Ergeta, Samegrelo-Zemo Svaneti, GRG	GRG3	257.8	42.3861	41.6803	1.0000	0.92	0.9200	4	11
Georgia	Zeda sameba, GRG	GRG4	538.1	41.7991	41.8671	0.8355	0.92	0.7687	4	11
Georgia	Kvaisi, South Ossetia, GRG	GRG5	1522.8	42.5365	43.6469	0.0161	0.25	0.0040	0	3
Georgia	Rustavi, Kvemo Kartli, GRG	GRG6	850.1	41.5611	45.1959	0.0562	0.33	0.0186	1	4
Georgia	Telavi, Kakheti, GRG	GRG7	958.6	41.9463	45.5036	0.0580	0.25	0.0145	1	3
Georgia	Lahili, Samegrelo-Zemo Svaneti, GRG	GRG8	1668.8	42.9998	42.6361	0.0748	0.42	0.0314	1	5
Georgia	Vabiskhevi, Borjomi nature Reserve, GRG	GRG9	1418.1	41.8049	43.1587	0.0291	0.33	0.0096	1	4
Turkey	Ilyaslar, Bolu, TKY	TKY1	1142.1	40.9807	32.3042	0.0277	0.25	0.0069	0	3
Turkey	Buyukcakirman, Erzincan, TKY	TKY2	948.1	39.7756	39.6455	0.0446	0.17	0.0076	0	2
Turkey	Karakoy, Denizli, TKY	TKY3	681.1	38.2045	28.8984	0.0106	0.08	0.0008	0	1
Turkey	Gercam Mahallesi, Denizli, TKY	TKY4	699.8	37.2487	28.9424	0.0191	0.08	0.0015	0	1
Turkey	Sagrak Koyu, Isparta, TKY	TKY5	1191.7	37.5629	30.9858	0.0162	0.17	0.0028	0	2
Turkey	Cesmelisebil, Konya, TKY	TKY6	1020.8	38.6178	32.5459	0.0047	0.17	0.0008	0	2
Turkey	Isikkaya Mahallesi, Adana, TKY	TKY7	1071.5	37.7021	35.5561	0.0090	0.17	0.0015	0	2
Turkey	Kaymakli, Erzican, TKY	TKY8	1468.9	39.3012	38.8740	0.0000	0	0.0000	0	0
Turkey	kale Koyu, Rize, TKY	TKY9	1555.5	40.8313	40.9175	0.1111	0.67	0.0744	1	8
Turkey	Merkez mahallesi, Sinop, TKY	TKY10	510.6	41.8737	34.8829	0.2507	0.58	0.1454	2	7
Turkey	Sulek Koyu, Bartin, TKY	TKY11	671.6	41.4798	32.2462	0.1085	0.58	0.0629	1	7
Turkey	Sayalar Mahallesi, Istanbul, TKY	TKY12	73.9	41.3480	28.1592	0.6987	0.67	0.4681	3	8
Turkey	Camliova Mahallesi, Antalya, TKY	TKY13	281.3	36.3904	29.4776	0.7620	0.75	0.5715	3	9
Turkey	Agackoy Koyu, Canakkale, TKY	TKY14	256.9	39.7925	26.5693	0.2326	0.5	0.1163	2	6
Turkey	Dogla Mahallesi, Bursa, TKY	TKY15	181.6	40.2971	28.2612	0.3818	0.58	0.2214	2	7
Turkey	Kaleici Mahallesi, Istanbul, TKY	TKY16	73.9	41.1630	28.4370	0.6987	0.67	0.4681	3	8
Turkey	Camkoy Koyu, Canakkale, TKY	TKY17	161.1	40.0032	26.8000	0.3284	0.5	0.1642	2	6
Turkey	Musellim Koyu, Kirklareli, TKY	TKY18	134.6	41.4353	27.2175	0.0751	0.5	0.0376	1	6
Turkey	Canakca Mahllesi, Istanbul, TKY	TKY19	73.9	41.2539	28.4919	0.6987	0.67	0.4681	3	8
Turkey	Imamlar Mahallesi, Sakarya, TKY	TKY20	77.3	41.0719	30.4255	0.9540	0.83	0.7918	4	10

						Pst				
Region and Country	Location	Code	Elevation (m)	Latitude (°N)	Longitude (°E)	MINF (A)	Fav-month (F)	Favourable index (A x F)	Risk score	Month
Turkey	Haydarlar Mahallesi, Ankara, TKY	TKY21	1077.3	40.2720	31.9416	0.0103	0.08	0.0008	0	1
Turkey	Ezelter Koyu, Giresun, TKY	TKY22	935.6	40.7564	38.2038	0.2332	0.5	0.1166	2	6
Turkey	Ortaklar Koyu, Rize, TKY	TKY23	1555.5	40.7731	40.8428	0.1111	0.67	0.0744	1	8
Turkey	Esmepinar Koyu, Erzican, TKY	TKY24	1929.8	39.9022	39.9177	0.0077	0.17	0.0013	0	2
Turkey	Yolgecen, Artvin, TKY	TKY25	852.7	41.3034	41.2899	0.6660	0.83	0.5528	3	10
Turkey	Kaplanli Kyo, Afyonkarahisar, TKY	TKY26	1230.5	38.9176	30.5793	0.0217	0.17	0.0037	0	2
Turkey	Kokulupinar Koyu, Agri, TKY	TKY27	2009.8	39.9527	42.7302	0.0044	0.08	0.0004	0	1
Turkey	Sekirhan, Diyarbakir, TKY	TKY28	1475.9	38.5405	40.9724	0.0070	0.25	0.0017	0	3
Turkey	Bulduk Mahallesi, Konya, TKY	TKY29	1031.4	39.0201	32.7107	0.0042	0.08	0.0003	0	1
Turkey	Yeni Mahallesi, Denizli, TKY	TKY30	681.1	38.0576	28.8435	0.0106	0.08	0.0008	0	1
Turkey	Ceceli Mahallesi, Kahramanmaras, TKY	TKY31	799.9	37.3449	36.8415	0.0245	0.25	0.0061	0	3
Turkey	Yagmurlar Koyu, Kutaha, TKY	TKY32	1111.7	39.0833	29.6125	0.0257	0.17	0.0044	0	2
Turkey	Eskigomu Koyu, Afyonkarahisar, TKY	TKY33	1143.1	39.0833	30.9748	0.0239	0.17	0.0041	0	2
Turkey	Adakoy Koyu, Zonguldak, TKY	TKY34	671.6	41.4353	32.1833	0.1085	0.58	0.0629	1	7
Turkey	Yonkali Koyu, Corum, TKY	TKY35	1026.5	40.7731	34.2048	0.0135	0.17	0.0023	0	2
Turkey	Gundogan Mahallesi, Ankara, TKY	TKY36	1000.7	39.4625	32.2492	0.0079	0.08	0.0006	0	1
Turkey	Dereli Koyu, Bartin, TKY	TKY37	404.9	41.6491	32.8205	0.5554	0.67	0.3721	3	8
Turkey	Ahlatcik Koyu, Corum, TKY	TKY38	1026.5	40.7731	34.2707	0.0135	0.17	0.0023	0	2
Turkey	Yaylakent Koyu, Tokat, TKY	TKY39	1076.9	40.1378	36.0725	0.0233	0.17	0.0040	0	2
Turkey	Akoren, Mengen, TKY	TKY40	40.357381	41.0043	32.0315	0.3614	0.75	0.2710	2	9
	**Proportion of sites with infection index**	**0.983**								
**Southwest Asia (50)**										
Egypt	Unnamed, Hala'ib Triangle, EGY	EGY1	152	22.5745	35.9516	0.0000	0	0.0000	0	0
Egypt	Unnamed, Qesm Marsa Alam, EGY	EGY2	283.3	25.5435	34.4575	0.0000	0	0.0000	0	0
Egypt	Qism ras Ghareb, Red Sea, EGY	EGY3	321.8	27.8206	33.1611	0.0000	0	0.0000	0	0
Egypt	Qesm Nwebaa, South Sinai, EGY	EGY4	726.4	29.1531	34.5454	0.0000	0	0.0000	0	0
Egypt	Arish, North Sinai, EGY	EGY5	194.9	30.9786	33.8203	0.0000	0	0.0000	0	0
Egypt	Kom-El-Farag, El Beheira, EGY	EGY6	29.1	30.9786	30.0190	0.0000	0.08	0.0000	0	1

						Pst				
Region and Country	Location	Code	Elevation (m)	Latitude (°N)	Longitude (°E)	MINF (A)	Fav-month (F)	Favourable index (A x F)	Risk score	Month
Egypt	Qism Moury Matrouch, Matrough, EGY	EGY7	117.5	30.6956	27.6679	0.0000	0	0.0000	0	0
Egypt	Qesm Sidi Brani, Matrough, EGY	EGY8	28	31.5046	25.9101	0.2412	0	0.0000	0	0
Egypt	Qesm Sidi Brani 1, Matrough, EGY	EGY9	-26.1	29.0187	27.3164	0.0000	0.5	0.0000	0	6
Egypt	Qesm Al Wahat Al Khargah 1, New Valley, E	EGY10	224.2	26.8643	31.2594	0.0000	0	0.0000	0	0
Egypt	Qesm Al Wahat Al Khargah 2, New Valley, E	EGY11	254.3	22.8785	32.3701	0.0000	0	0.0000	0	0
Egypt	Abu ar Rish Basri, Red Sea, EGY	EGY12	252.6	24.2480	32.9853	0.0000	0	0.0000	0	0
Egypt	Qesm Al Wahat Ad Dakhlah 1, New Valley, E	EGY13	218.6	22.9797	29.6894	0.0000	0	0.0000	0	0
Egypt	Qesm Hurghada 1, Red Sea, EGY	EGY14	452.5	26.9843	33.2957	0.0000	0	0.0000	0	0
Egypt	Qesm Sham Ash Sheikh, South Sinai, EGY,	EGY15	418.2	28.3320	34.1197	0.0000	0	0.0000	0	0
Egypt	Ataqah 1, Suez, EGY	EGY16	341.1	29.4525	32.3838	0.0000	0	0.0000	0	0
Egypt	Qesm Hurghada 2, Red Sea, EGY	EGY17	464.1	27.3776	33.1336	0.0000	0	0.0000	0	0
Egypt	Unanmed, Red Sea, EGY	EGY18	226.4	27.9857	31.1396	0.0000	0	0.0000	0	0
Egypt	Ataqah 2, Suez, EGY	EGY19	341.1	29.3544	32.1833	0.0000	0	0.0000	0	0
Egypt	Shata, Damietta, EGY	EGY20	2.4	31.4522	31.8946	0.0095	0	0.0000	0	0
Iraq	Aghajah Mashad, Tooz, IQ	IRQ1	503.8	35.0084	44.6675	0.0041	0.08	0.0003	0	1
Iraq	Unnamed, Ana, IQ	IRQ2	339.8	33.6292	41.8990	0.0000	0	0.0000	0	0
Iraq	Unnamed, RTE France, KT	IRQ3	121.5	29.7415	47.3482	0.0124	0.08	0.0010	0	1
Iraq	Unnamed, Najaf, IQ	IRQ4	330.5	30.2363	44.0083	0.0060	0.08	0.0005	0	1
Iraq	Unnamed, Ramadi, IQ	IRQ5	84.5	33.0418	43.4370	0.0000	0	0.0000	0	0
Israel	Givat Nili, Har Alexander, IS	IS1	176.9	32.5408	35.0425	0.0422	0.25	0.0105	1	3
Israel	Ohad, Eshkol, IS	IS2	104.5	31.2349	34.4493	0.0102	0.17	0.0017	0	2
Lebanon	Mazraat Matariyet Jbaa, Maghdouche', LB	Lb1	258.8	33.5171	35.3941	0.9285	0.58	0.5385	3	7
Oman	Fajri, Al Batinah, OM	OM1	625.3	23.4970	57.1910	0.0000	0	0.0000	0	0
Oman	Unnamed, Ash Sharqiyah, South, OM	OM2	98.9	22.2418	59.5640	0.0000	0	0.0000	0	0
Oman	Unnamed, Dhofar, OM	OM3	423.8	17.4906	54.9058	0.0000	0	0.0000	0	0
Oman	Hai;at La'mia, Al Wusta, OM	OM4	114.1	20.9753	56.0044	0.0000	0	0.0000	0	0
Saudi Arab	Tawala, Tabuk, SA	SA1	233.1	25.4567	37.2837	0.0000	0	0.0000	0	0
Saudi Arab	Unnamed 1, Tabuk, SA	SA2	516.8	28.2788	35.4380	0.0000	0	0.0000	0	0
Saudi Arab	Unnamed 2, Tabuk, SA	SA3	422.8	24.9796	37.5474	0.0000	0	0.0000	0	0

Region and Country	Location	Code	Elevation (m)	Latitude (°N)	Longitude (°E)	*Pst* MINF (A)	Fav-month (F)	Favourable index (A x F)	Risk score	Month
Saudi Arab	Unnamed, Al Qunfudhah, SA	SA4	578.5	19.5738	41.3706	0.0000	0	0.0000	0	0
Saudi Arab	Shuwaq, Tabuk, SA	SA5	431.8	27.4630	36.4048	0.0000	0	0.0000	0	0
Saudi Arab	Unanmmed, Forest area, SA	SA6	798	30.7286	39.4820	0.0000	0	0.0000	0	0
Saudi Arab	Unnamed, Northern Borders, SA	SA7	576.8	29.6269	41.8111	0.0000	0	0.0000	0	0
Saudi Arab	Unanmed, Al Nufud Al Kabir, SA	SA8	546.2	26.8397	45.6783	0.0013	0.08	0.0001	0	1
Saudi Arab	Unnamed, Al Aflaj, SA	SA9	448.9	21.1829	47.3482	0.0000	0	0.0000	0	0
Saudi Arab	Al Shinayin, Abu Dhabi, UAE	SA10	80.1	23.7009	54.2915	0.0000	0	0.0000	0	0
Saudi Arab	Unnamed, Al Ahsa, SA	SA11	87.6	25.4591	50.1607	0.0000	0	0.0000	0	0
Syria	Babdh, Jableh, SY	SY1	382.5	35.2578	36.0533	0.0530	0.42	0.0223	1	5
Syria	Khirbet Ghazaleh, Daraa, SY	SY2	710.7	32.7074	36.1631	0.0379	0.17	0.0064	0	2
Syria	Palmyra, SY	SY3	639.6	34.8440	38.1846	0.0379	0.17	0.0064	0	2
Yemen	Karad, Ad Dali, YM	YM1	1424.2	13.8098	44.5787	0.0000	0	0.0000	0	0
Yemen	Samarma, Al Mahrah, YM	YM2	440.1	15.2985	50.9507	0.0000	0	0.0000	0	0
Yemen	Unnamed, Hadhramaut, YM	YM3	623.8	17.5325	48.2261	0.0000	0	0.0000	0	0
Yemen	Al Harajah, Hajjah, YM	YM4	106.9	15.9756	42.9966	0.0000	0	0.0000	0	0
	**Proportion of sites with infection index**	**0.24**								
**Adjoining European countries (53)**										
Albania	Sheshi, Tepelene, ALB	ALB1	315.4	40.3474	19.9150	0.6766	0.83	0.5616	3	10
Bulgaria	Zhitosvyat, Karnobat, BL	BL1	235.1	42.4915	26.9806	0.0124	0.25	0.0031	0	3
Bulgaria	Krupen, Kavarna, BL	BL2	99.7	43.5517	28.2331	0.1583	0.58	0.0918	1	7
Bulgaria	Prismenovo, Primorsko, BL	BL3	149.2	42.2317	27.6178	0.4070	0.67	0.2727	2	8
Bulgaria	Kartipanya, Dryanovo, BL	BL4	479.1	42.9274	25.4645	0.1621	0.42	0.0681	1	5
Bulgaria	Manastirishte, Hayredin, BL	BL5	143.2	43.5517	23.6408	0.0698	0.33	0.0230	1	4
Bulgaria	Pomen, Dve mongili, BL	BL6	300.1	43.4879	25.9699	0.0430	0.42	0.0181	1	5
Bulgaria	Byalkovtsi, Elena, BL	BL7	344.5	42.9435	26.0138	0.0240	0.17	0.0041	0	2
Bulgaria	Trakia, Opan, BL	BL8	322.5	42.1992	25.6183	0.0136	0.17	0.0023	0	2
Bulgaria	Byal Kladenets, Stambolovo, BL	BL9	317.7	41.6433	25.6183	0.0155	0.25	0.0039	0	3
Bulgaria	Lyahovo, Balchik, BL	BL10	74.7	43.4251	28.0274	0.6336	0.75	0.4752	3	9

Region and Country	Location	Code	Elevation (m)	Latitude (°N)	Longitude (°E)	*Pst* MINF (A)	Fav-month (F)	Favourable index (A x F)	Risk score	Month
Croatia	Ridane, CR	CR1	404.2	43.9304	16.2676	0.4712	0.83	0.3911	3	10
Croatia	Baska, Opcina, CR	CR2	252.2	44.9034	14.7734	0.8285	1	0.8285	4	12
Greece	Doumpia, Anthemountas, GR	GR1	277.4	40.5147	23.3393	0.1410	0.42	0.0592	1	5
Greece	Kokkinaras, Koropi, GR	GR2	74.2	37.9104	23.9326	0.7091	0.75	0.5318	3	9
Greece	Gergeri, Rouvas, GR	GR3	172.3	35.1747	24.9433	0.9286	0.75	0.6965	3	9
Greece	Dafniotissa, Amaliada, GR	GR4	34	37.8410	21.4717	0.7937	0.83	0.6588	3	10
Greece	Plagia, Acheloos, GR	GR5	785.4	39.2331	21.4277	0.3082	0.42	0.1294	2	5
Greece	Paleochori, Sperchiada, GR	GR6	630.5	38.8406	21.9551	0.2339	0.58	0.1356	2	7
Italy	Ai Boschi Castelminio, Treviso, IT	IT1	288.6	45.6456	12.0049	0.3160	0.92	0.2907	2	11
Italy	Ostellato, Ferrara, IT	IT2	51.5	44.7163	11.9170	0.1724	0.58	0.1000	1	7
Italy	Sassocorvaro, Pesaro, IT	IT3	392.9	43.7719	12.4883	0.3410	0.67	0.2285	2	8
Italy	Campli, Teramo, IT	IT4	460.5	42.7156	13.6748	0.6597	0.75	0.4948	3	9
Italy	Acquaviva Collecroce, Campobasso, IT	IT5	395.8	41.8677	14.7295	0.4340	0.75	0.3255	3	9
Montenegro	Lipa, Cetinje, MN	MN1	839.6	42.5539	18.8603	0.4905	0.83	0.4071	3	10
Romania	Unnamed, Casimcea, RM	RM1	59.7	44.6851	28.3985	0.0407	0.25	0.0102	1	3
Romania	Valea Seman, Urlati, RM	RM2	503.6	45.0122	26.2892	0.0470	0.33	0.0155	1	4
Romania	Maneciu-Ungureni, Maneciu, RM	RM3	503.6	45.3220	26.0090	0.0470	0.33	0.0155	1	4
Romania	Unnamed, Bustuchin, RM	RM4	333.4	44.9656	23.7129	0.0766	0.42	0.0322	1	5
Romania	Piscu Nou, Piscu Vechi, RM	RM5	185.3	43.9224	23.1526	0.0775	0.33	0.0256	1	4
Romania	Valcelele de Sus, Valcele, RM	RM6	135.2	44.2932	24.5806	0.0384	0.42	0.0161	1	5
Romania	Runc, Zlatna, RM	RM7	636.5	46.1882	23.1965	0.0935	0.42	0.0393	1	5
Romania	Strugureni, Chiochis, RM	RM8	485.5	46.9810	24.2292	0.0455	0.42	0.0191	1	5
Romania	Cuza Voda, racovita, RM	RM9	121.3	45.3607	27.4372	0.0401	0.33	0.0132	1	4
Romania	Razim lake, RM	RM10	12.4	44.8021	29.1181	0.6419	0.42	0.2696	2	5
Romania	Podul Lung, Calarasi, MLD	RM11	158.4	47.2576	28.2502	0.0286	0.75	0.0214	1	9
Romania	Caltesti, Pietroasele, RM	RM12	296.6	45.1131	26.5913	0.0438	0.17	0.0075	0	2
Slovenia	Vedrijan, SL	SL1	778.1	46.0130	13.5430	0.2074	0.67	0.1389	2	8
Ukraine	Verkhni Stanivtsi, Chernivtsi Oblast, UKR	UKR1	408.5	48.2693	25.5696	0.4578	0.42	0.1923	2	5
Ukraine	Pavlivka, Odessa Oblast, UKR	UKR2	28.4	45.9596	29.5466	0.1365	0.67	0.0914	1	8

Region and Country	Location	Code	Elevation (m)	Latitude (°N)	Longitude (°E)	*Pst* MINF (A)	Fav-month (F)	Favourable index (A x F)	Risk score	Month
Ukraine	Creamian Peninsula, UKR	UKR3	222.2	44.6382	34.1334	0.2171	0.67	0.1455	2	8
Ukraine	Sevastopol 1, Crimean, UKR	UKR4	66.4	45.3838	33.8642	0.7011	0.58	0.4067	3	7
Ukraine	Sevastopol 2, Crimean, UKR	UKR5	10.8	46.4992	32.8315	0.3865	0.58	0.2242	2	7
Ukraine	Novohryhorivka, Kherson Oblast, UKR	UKR6	7.8	46.4083	35.0068	0.0773	0.75	0.0580	1	9
Ukraine	Yelyseivka, Zaporizhia Oblast, UKR	UKR7	44.6	46.9960	36.4570	0.0485	0.75	0.0364	1	9
Ukraine	Ahronomichne, Donetsk Oblast, UKR	UKR8	141.9	47.8582	38.1709	0.0410	0.42	0.0172	1	5
Ukraine	Shtormove, Luhansk Oblast, UKR	UKR9	127.5	49.0962	38.9179	0.0267	0.5	0.0134	1	6
Ukraine	Zavadivka, Kirovohrad Oblast, UKR	UKR10	154.2	48.0054	32.3042	0.0825	0.42	0.0347	1	5
Ukraine	Kunyche, Vinnytsia Oblast, UKR	UKR11	219.1	48.4882	28.9424	0.0987	0.33	0.0326	1	4
Ukraine	Potaptsi, Cherkasy Oblast, UKR	UKR12	140.1	49.8529	31.2275	0.0674	0.33	0.0222	1	4
Ukraine	Kalivka, Sumy Oblast, UKR	UKR13	167.1	52.0667	33.5786	0.0921	0.42	0.0387	1	5
Ukraine	Rohovchi, Khmelnytskyi Oblast, UKR	UKR14	244.9	50.0931	27.3823	0.2102	0.42	0.0883	1	5
Ukraine	Novi Sokoly, Kyiv Oblast, UKR	UKR15	132.8	51.1525	29.8872	0.0660	0.42	0.0277	1	5
	**Total number of sites or *mean***	**607**				***0.0933***	***0.2532***	***0.0558***	***0.6474***	***3.04***

Table S1. Elevations, latitudes, longitudes, monthly infection index (MIN*formis* f. sp. *tritici (Pst*) and the stem rust pathogen,
P. graminis f. sp. *tritici* (*Pgt*) in relation to barberry growth in Asia and a

			Pgt					Barberry				
Region and Country	Location	Code	MINF (B)	Fav-month (F)	Favourable index (B x F)	Risk score	Month	NHTT (D)	Fav-month (F)	Favourable growth index (D x F)	Suitabilty score	Months
**East Asia (250)**												
Bangladesh	Nalian Range, BD	BD1	0.1068	0.42	0.0448	1	5	0.0137	0.33	0.0045	0	4
Bangladesh	Rangamati, BD	BD2	0.1634	0.58	0.0948	1	7	0.0473	0.42	0.0199	0	5
Bangladesh	Genduram, BD	BD3	0.0536	0.50	0.0268	1	6	0.0332	0.42	0.0138	0	5
Bangladesh	Durgaour, BD	BD4	0.1475	0.42	0.0619	1	5	0.0102	0.25	0.0026	0	3
Bangladesh	Ati Ulain, BD	BD5	0.0920	0.33	0.0303	1	4	0.0232	0.33	0.0077	0	4
Bhutan	Wangdue Phodrang, BH	BH1	0.3315	0.33	0.1094	2	4	0.8407	1.00	0.8407	4	12
Bhutan	Samdrup Jongkhar, BH	BH2	0.0868	0.67	0.0581	1	8	0.0553	0.50	0.0277	0	6
Bhutan	Gasa, BH	BH3	0.1887	0.33	0.0623	1	4	0.8383	1.00	0.8383	4	12
Bhutan	Lhuntse, BH	BH4	0.1881	0.33	0.0621	1	4	0.7762	1.00	0.7762	4	12
Bhutan	Kangpar, BH	BH5	0.1845	0.67	0.1236	2	8	0.4759	1.00	0.4759	2	12
Bhutan	Xigate, BH	BH6	0.2451	0.33	0.0809	1	4	0.8477	1.00	0.8477	4	12
Bhutan	Putao 2, BH	BH7	0.2861	0.42	0.1202	2	5	0.5732	1.00	0.5732	3	12
Bhutan	Putao 3, BH	BH8	0.1320	0.58	0.0766	1	7	0.1026	0.58	0.0598	0	7
Bhutan	Daifam, BH	BH9	0.0863	0.58	0.0501	1	7	0.0603	0.58	0.0352	0	7
China	Litang, Sichuan, CH	CH1	0.0418	0.08	0.0033	0	1	0.7599	1.00	0.7599	4	12
China	Batang 1, Sichuan, CH	CH2	0.0443	0.25	0.0111	1	3	0.7359	1.00	0.7359	4	12
China	Kangding, Sichuan, CH	CH3	0.0525	0.17	0.0089	1	2	0.8197	1.00	0.8197	4	12
China	Shawan 1, Sichuan, CH	CH4	0.3477	0.83	0.2886	2	10	0.4160	0.75	0.3120	2	9
China	Suijiang, Yunnan, CH	CH5	0.3722	0.75	0.2791	2	9	0.4639	0.83	0.3866	2	10
China	Dechang, Sichuan, CH	CH6	0.1359	0.50	0.0680	1	6	0.4602	1.00	0.4602	2	12
China	Huangling, Shaanxi, CH	CH7	0.0456	0.33	0.0150	1	4	0.4532	1.00	0.4532	2	12
China	Jiaokou, Shaanxi, CH	CH8	0.0287	0.17	0.0049	0	2	0.4143	1.00	0.4143	2	12
China	Shennonhjia, Hubei, CH	CH9	0.0377	0.42	0.0158	1	5	0.4751	1.00	0.4751	2	12
China	Kaixian, Chongqing, CH	CH10	0.1516	0.58	0.0879	1	7	0.4576	0.83	0.3813	2	10
China	Hejiang, Sichuan, CH	CH11	0.2794	0.83	0.2319	2	10	0.3538	0.75	0.2654	1	9
China	Alxa, Inner Mongolia, CH	CH12	0.0507	0.42	0.0213	1	5	0.4402	1.00	0.4402	2	12

			Pgt					Barberry				
Region and Country	Location	Code	MINF (B)	Fav-month (F)	Favourable index (B x F)	Risk score	Month	NHTT (D)	Fav-month (F)	Favourable growth index (D x F)	Suitabilty score	Months
China	Tianzhu, Gansu, CH	CH13	0.0288	0.17	0.0049	0	2	0.5637	1.00	0.5637	3	12
China	Guinan, Qinghai, CH	CH14	0.0045	0.08	0.0004	0	1	0.5866	1.00	0.5866	3	12
China	Nanzhao, Henan, CH	CH15	0.0292	0.42	0.0122	1	5	0.4372	0.83	0.3644	2	10
China	Zhenxiong, Yunnan, CH	CH16	0.2612	0.75	0.1959	2	9	0.4295	0.92	0.3937	2	11
China	Fengqing, Yunnan, CH	CH17	0.1159	0.67	0.0776	1	8	0.2181	1.00	0.2181	1	12
China	Batang 2, Sichuan, CH	CH18	0.0443	0.25	0.0111	1	3	0.7359	1.00	0.7359	4	12
China	Daguan, Yunnan, CH	CH19	0.3170	0.75	0.2377	2	9	0.4133	0.83	0.3444	2	10
China	Danba, Sichuan, CH	CH20	0.0117	0.25	0.0029	0	3	0.7751	1.00	0.7751	4	12
China	Dayi, Sichuan, CH	CH21	0.4200	0.58	0.2436	2	7	0.6116	1.00	0.6116	3	12
China	Shawan 2, Sicuan, CH	CH22	0.3477	0.83	0.2886	2	10	0.4160	0.75	0.3120	2	9
China	Junlian xian, Sichuan, CH	CH23	0.3170	0.75	0.2377	2	9	0.4133	0.83	0.3444	2	10
China	Banan, Chongqing, CH	CH24	0.2496	0.75	0.1872	2	9	0.3907	0.75	0.2930	1	9
China	Xixia, Henan, CH	CH25	0.0348	0.25	0.0087	1	3	0.4459	0.83	0.3716	2	10
China	Taibai, Shaanxi, CH	CH26	0.0706	0.33	0.0233	1	4	0.5121	1.00	0.5121	3	12
China	Wangyi, Shaanxi, CH	CH27	0.0590	0.33	0.0195	1	4	0.4397	0.92	0.4030	2	11
China	Maiji, Gansu, CH	CH28	0.0393	0.42	0.0165	1	5	0.5060	1.00	0.5060	3	12
China	Zhenba, Shaanxi, CH	CH29	0.0968	0.67	0.0649	1	8	0.4976	0.92	0.4562	2	11
China	Tewo, Gansu, CH	CH30	0.0124	0.17	0.0021	0	2	0.6640	1.00	0.6640	3	12
China	Jiuzhi,Qinghai, CH	CH31	0.0004	0.08	0.0000	0	1	0.6432	1.00	0.6432	3	12
China	Wudu, Gansu, CH	CH32	0.0775	0.33	0.0256	1	4	0.5256	1.00	0.5256	3	12
China	Jiulong,Sichuan, CH	CH33	0.1510	0.50	0.0755	1	6	0.7474	1.00	0.7474	4	12
China	Xinlong, Sichuan, CH	CH34	0.0012	0.08	0.0001	0	1	0.6911	1.00	0.6911	3	12
China	Yanyuan, Sichuan, CH	CH35	0.1325	0.50	0.0662	1	6	0.5201	1.00	0.5201	3	12
China	Huize, Yunnan, CH	CH36	0.1017	0.50	0.0508	1	6	0.4360	1.00	0.4360	2	12
China	Jinsha, Guizhou, CH	CH37	0.1709	0.83	0.1419	2	10	0.4048	0.83	0.3374	2	10
China	Mohe, Heilongjiang, CH	CH38	0.0659	0.42	0.0277	1	5	0.3088	0.75	0.2316	1	9
China	Jianning, Fujian, CH	CH39	0.1763	0.58	0.1023	2	7	0.3227	0.75	0.2420	1	9
China	Yuxian, Yunnan, CH	CH40	0.1159	0.67	0.0776	1	8	0.2181	1.00	0.2181	1	12
China	Putao 4, Mym	CH41	0.0479	0.42	0.0201	1	5	0.1699	0.67	0.1133	1	8

			Pgt					Barberry				
Region and Country	Location	Code	MINF (B)	Fav-month (F)	Favourable index (B x F)	Risk score	Month	NHTT (D)	Fav-month (F)	Favourable growth index (D x F)	Suitabilty score	Months
China	Maizhokunggar, Lhasa, CH	CH42	0	0.00	0.0000	0	0	0.6350	1.00	0.6350	3	12
China	Dengqen, Tibet, CH	CH43	0	0.00	0.0000	0	0	0.5243	0.92	0.4806	2	11
China	Sunan, Gansu, CH	CH44	0	0.00	0.0000	0	0	0.5336	1.00	0.5336	3	12
China	Tahe, Heilongjiang, CH	CH45	0.0597	0.42	0.0251	1	5	0.2928	0.75	0.2196	1	9
China	Yakeshi, Inner Mongolis, CH	CH46	0.0207	0.25	0.0052	0	3	0.3222	0.75	0.2416	1	9
China	Jinshantun, Heilongjiang, CH	CH47	0.0314	0.33	0.0103	1	4	0.3021	0.75	0.2265	1	9
China	Zalantun, Inner Mongolia, CH	CH48	0.0081	0.25	0.0020	0	3	0.3324	0.83	0.2770	1	10
China	Zamtang, Sicuana, CH	CH49	0.0008	0.08	0.0001	0	1	0.7225	1.00	0.7225	4	12
China	Wuxi, Chongqing, CH	CH50	0.0751	0.58	0.0435	1	7	0.5251	1.00	0.5251	3	12
China	Yongding, Huanan, CH	CH51	0.1895	0.75	0.1421	2	9	0.4483	0.83	0.3736	2	10
China	Xiushui, Jiangxi, CH	CH52	0.1439	0.75	0.1079	2	9	0.3961	0.75	0.2971	1	9
China	Yingde, Guangdong, CH	CH53	0.1633	0.75	0.1225	2	9	0.2245	0.67	0.1497	1	8
China	Tian'e, Guangxi, CH	CH54	0.1505	0.75	0.1129	2	9	0.2829	0.75	0.2121	1	9
China	Baiyu, Sichuan, CH	CH55	0.0050	0.08	0.0004	0	1	0.6821	1.00	0.6821	3	12
China	Alxa Zuoqi, Inner Mongolia, CH	CH56	0.0200	0.25	0.0050	0	3	0.3652	1.00	0.3652	2	12
China	Biru, Tibet, CH	CH57	0	0.00	0.0000	0	0	0.5826	1.00	0.5826	3	12
China	Nagqu 1, Tibet, CH	CH58	0	0.00	0.0000	0	0	0.5374	1.00	0.5374	3	12
China	Nagqu 2, Tibet, CH	CH59	0	0.00	0.0000	0	0	0.5519	1.00	0.5519	3	12
China	Gyaca 1, Tibet, CH	CH60	0.0023	0.08	0.0002	0	1	0.7335	1.00	0.7335	4	12
China	Nangxian, Tibet, CH	CH61	0.1165	0.25	0.0291	1	3	0.8627	1.00	0.8627	4	12
China	Jiulong, Sichuan, CH	CH62	0.1165	0.25	0.0291	1	3	0.8627	1.00	0.8627	4	12
China	Wenxian, Gansu, CH	CH63	0.1186	0.25	0.0296	1	3	0.5599	1.00	0.5599	3	12
China	Wenchuan, Sichuan, CH	CH64	0.1020	0.50	0.0510	1	6	0.7178	1.00	0.7178	4	12
China	Barkam, Sichuan, CH	CH65	0.1926	0.42	0.0809	1	5	0.7414	1.00	0.7414	4	12
China	Hongya, Sichuan,CH	CH66	0	0.00	0.0000	0	0	0.4796	0.83	0.3997	2	10
China	Hanyuan, Sichuan, CH	CH67	0.4291	0.83	0.3561	3	10	0.7172	1.00	0.7172	4	12
China	Butuo 1, Sichuan, CH	CH68	0.2307	0.42	0.0969	1	5	0.8320	1.00	0.8320	4	12
China	Butuo 2, Sichuan, CH	CH69	0.0684	0.25	0.0171	1	3	0.5108	1.00	0.5108	3	12
China	Muchuan, Sichuan, CH	CH70	0.1497	0.50	0.0748	1	6	0.4639	0.83	0.3866	2	10

			Pgt					Barberry				
Region and Country	Location	Code	MINF (B)	Fav-month (F)	Favourable index (B x F)	Risk score	Month	NHTT (D)	Fav-month (F)	Favourable growth index (D x F)	Suitabilty score	Months
China	Leibo, Sichuan, CH	CH71	0.3722	0.75	0.2791	2	9	0.4692	1.00	0.4692	2	12
China	Luquan, Yunnan, CH	CH72	0.3384	0.58	0.1963	2	7	0.3593	1.00	0.3593	2	12
China	Lanping, Yunnan, CH	CH73	0.0704	0.58	0.0408	1	7	0.5882	1.00	0.5882	3	12
China	Huili, Sichuan, CH	CH74	0.2310	0.50	0.1155	2	6	0.3969	1.00	0.3969	2	12
China	Dayao, Yunnan, CH	CH75	0.1093	0.58	0.0634	1	7	0.3661	1.00	0.3661	2	12
China	Mainling, Tibet, CH	CH76	0.1010	0.58	0.0586	1	7	0.8574	1.00	0.8574	4	12
China	Jinchuan, Sichuan, CH	CH77	0.1272	0.33	0.0420	1	4	0.7737	1.00	0.7737	4	12
China	Zhuanglang, Gansu, CH	CH78	0.0128	0.17	0.0022	0	2	0.4913	1.00	0.4913	2	12
China	Zhenyuan, Gansu, CH	CH79	0.0320	0.42	0.0135	1	5	0.4552	1.00	0.4552	2	12
China	Dingbian, Shaanxi, CH	CH80	0.0479	0.25	0.0120	1	3	0.4254	1.00	0.4254	2	12
China	Qilian, Qinghai, CH	CH81	0.0057	0.08	0.0005	0	1	0.4420	0.92	0.4052	2	11
China	Nanbu, Sichuan, CH	CH82	0.1385	0.58	0.0803	1	7	0.3922	0.75	0.2941	1	9
China	Ulan 2, Qinghai, CH	CH83	0.0008	0.08	0.0001	0	1	0.5257	0.92	0.4819	2	11
China	Jomdo, Tibet, CH	CH84	0	0.00	0.0000	0	0	0.6230	1.00	0.6230	3	12
China	Wenchuan 2, Sichuan, CH	CH85	0.3032	0.42	0.1274	2	5	0.6629	1.00	0.6629	3	12
China	Pengzhou, Sichuan, CH	CH86	0.4660	0.67	0.3122	3	8	0.5667	1.00	0.5667	3	12
China	Mianzhu, Sichuan, CH	CH87	0.3231	0.67	0.2165	2	8	0.4599	0.83	0.3832	2	10
China	Pingshan 2, Sichuan, CH	CH88	0.2702	0.75	0.2026	2	9	0.3755	0.75	0.2816	1	9
China	Suijiang 2, Yunnan, CH	CH89	0.3722	0.75	0.2791	2	9	0.4639	0.83	0.3866	2	10
China	Shuifu, Yunan, CH	CH90	0.3170	0.75	0.2377	2	9	0.4133	0.83	0.3444	2	10
China	Tewo 2, Gansu, CH	CH91	0	0.00	0.0000	0	0	0.0087	0.25	0.0022	0	3
China	Meigu 2, Sichuan, CH	CH92	0.2844	0.58	0.1650	2	7	0.5377	1.00	0.5377	3	12
China	Ebian, Sichuan, CH	CH93	0.3745	0.67	0.2509	2	8	0.5170	1.00	0.5170	3	12
China	Mabian, Sichuan, CH	CH94	0.2844	0.58	0.1650	2	7	0.5377	1.00	0.5377	3	12
China	Wenchuan 3, Sichuan, CH	CH95	0.4200	0.58	0.2436	2	7	0.6116	1.00	0.6116	3	12
China	Ge'gyai, Tibet, CH	CH96	0	0.00	0.0000	0	0	0.4316	0.83	0.3597	2	10
China	Karakax, Xinjiang, CH	CH97	0.0064	0.25	0.0016	0	3	0.3295	0.83	0.2746	1	10
China	Hoboksar, Xinjiang, CH	CH98	0.0284	0.25	0.0071	0	3	0.2961	0.92	0.2714	1	11
China	Hejing, Xinjiang, CH	CH99	0.0021	0.08	0.0002	0	1	0.5265	0.92	0.4827	2	11

Region and Country	Location	Code	*Pgt* MINF (B)	*Pgt* Fav-month (F)	*Pgt* Favourable index (B x F)	*Pgt* Risk score	*Pgt* Month	Barberry NHTT (D)	Barberry Fav-month (F)	Barberry Favourable growth index (D x F)	Barberry Suitabilty score	Barberry Months
China	Kumul, Xinjiang, CH	CH100	0.0041	0.25	0.0010	0	3	0.3592	0.92	0.3293	2	11
China	Ruoqiang, Xinjiang, CH	CH101	0.0092	0.08	0.0007	0	1	0.5544	1.00	0.5544	3	12
China	Zhidoi, Qinghai, CH	CH102	0	0.00	0.0000	0	0	0.3841	0.75	0.2881	1	9
China	Shuanghu, Tibet, CH	CH103	0.0019	0.08	0.0002	0	1	0.5076	0.92	0.4653	2	11
China	Zhengxiangbaiqi, Inner Mongolia, CH	CH104	0.0037	0.17	0.0006	0	2	0.3340	1.00	0.3340	2	12
China	Urad Houqi, Inner Mongolia, CH	CH105	0.0079	0.17	0.0013	0	2	0.3531	1.00	0.3531	2	12
China	Aba, Sichuan, CH	CH106	0.0409	0.25	0.0102	1	3	0.6079	1.00	0.6079	3	12
China	Baoji2, Shaanxi, CH	CH107	0.1465	0.42	0.0615	1	5	0.4017	1.00	0.4017	2	12
China	Bazhong, Sichuan, CH	CH108	0.2204	0.67	0.1477	2	8	0.3541	0.75	0.2656	1	9
China	Chengxian, Gansu, CH	CH109	0.1100	0.42	0.0462	1	5	0.4139	1.00	0.4139	2	12
China	Dali, Yunan, CH	CH110	0.1224	0.33	0.0404	1	4	0.3260	1.00	0.3260	2	12
China	Degen, Yunan, CH	CH111	0.0831	0.42	0.0349	1	5	0.1729	1.00	0.1729	1	12
China	Deyang, Sichuan, CH	CH112	0.3648	0.67	0.2444	2	8	0.3685	0.83	0.3071	2	10
China	Dinxi, Gansu, CH	CH113	0.0564	0.33	0.0186	1	4	0.3891	1.00	0.3891	2	12
China	Fengxian, Shaanxi, CH	CH114	0.1519	0.42	0.0638	1	5	0.4227	1.00	0.4227	2	12
China	Gnagu, Gansu, CH	CH115	0.0789	0.33	0.0261	1	4	0.4088	1.00	0.4088	2	12
China	Gannan, Gansu, CH	CH116	0.0383	0.25	0.0096	1	3	0.3862	1.00	0.3862	2	12
China	Ganzi, Sichuan, CH	CH117	0.0838	0.25	0.0210	1	3	0.6355	1.00	0.6355	3	12
China	Guangyuan, Sichuan, CH	CH118	0.2229	0.67	0.1494	2	8	0.3814	0.83	0.3178	2	10
China	Haixi, Qinghai, CH	CH119	0.0093	0.08	0.0007	0	1	0.4121	1.00	0.4121	2	12
China	Hanzong, Shaanxi, CH	CH120	0	0.00	0.0000	0	0	0.6982	1.00	0.6982	3	12
China	Huixian, Gansu, CH	CH121	0.1208	0.33	0.0399	1	4	0.4152	1.00	0.4152	2	12
China	Lueyang, Shaanxi, CH	CH122	0.1220	0.42	0.0513	1	5	0.3826	1.00	0.3826	2	12
China	Kunming, Yunan, CH	CH123	0.0539	0.42	0.0226	1	5	0.2525	1.00	0.2525	1	12
China	Lanzhou, Gansu, CH	CH124	0.0383	0.25	0.0096	1	3	0.3862	1.00	0.3862	2	12
China	Linagdang, Gansu, CH	CH125	0.1208	0.33	0.0399	1	4	0.4152	1.00	0.4152	2	12
China	Liangshun, Sichuan, CH	CH126	0.1594	0.33	0.0526	1	4	0.4017	1.00	0.4017	2	12
China	Lijiang, Yunan, CH	CH127	0.1333	0.25	0.0333	1	3	0.4059	1.00	0.4059	2	12
China	Linxia, Gansu, CH	CH128	0.0599	0.33	0.0198	1	4	0.4227	1.00	0.4227	2	12

Region and Country	Location	Code	*Pgt* MINF (B)	Fav-month (F)	Favourable index (B x F)	Risk score	Month	Barberry NHTT (D)	Fav-month (F)	Favourable growth index (D x F)	Suitabilty score	Months
China	Lixian, Gansu, CH	CH129	0.0789	0.33	0.0261	1	4	0.4088	1.00	0.4088	2	12
China	Longnan, Gansu, CH	CH130	0.1906	0.42	0.0800	1	5	0.4565	1.00	0.4565	2	12
China	Maijigen, Gansu, CH	CH131	0.1098	0.42	0.0461	1	5	0.4125	1.00	0.4125	2	12
China	Mianyang, Sichuan, CH	CH132	0.2308	0.67	0.1546	2	8	0.3176	0.75	0.2382	1	9
China	Panlogjhen, Shaanxi, CH	CH133	0.1465	0.42	0.0615	1	5	0.4017	1.00	0.4017	2	12
China	Panzihua, Sichuan, CH	CH134	0.1186	0.33	0.0391	1	4	0.2760	1.00	0.2760	1	12
China	Qiangyang2, Sichuan, CH	CH135	0.1220	0.42	0.0513	1	5	0.3826	1.00	0.3826	2	12
China	Qingshui, Gansu, CH	CH136	0.1071	0.42	0.0450	1	5	0.4110	1.00	0.4110	2	12
China	Shannan, Tibet, CH	CH137	0.0018	0.08	0.0001	0	1	0.5908	1.00	0.5908	3	12
China	Taibai2, Shaanxi, CH	CH138	0.1465	0.42	0.0615	1	5	0.4017	1.00	0.4017	2	12
China	Tianshui, Gansu, CH	CH139	0.0810	0.33	0.0267	1	4	0.4011	1.00	0.4011	2	12
China	Wangchuamjen, Gansu, CH	CH140	0.0965	0.33	0.0318	1	4	0.4081	1.00	0.4081	2	12
China	Yaan, Sichuan, CH	CH141	0.5424	0.58	0.3146	3	7	0.4379	1.00	0.4379	2	12
China	Yuxi, Yunan	CH142	0.0504	0.42	0.0212	1	5	0.1724	1.00	0.1724	1	12
India	Deema Range, Uttarakhand, IN	IN1	0.0872	0.33	0.0288	1	4	0.0004	0.00	0.0000	0	0
India	Champawat,UK	IN2	0.1299	0.33	0.0429	1	4	0.2256	0.75	0.1692	1	9
India	Lunglei, Mizoram,IN	IN3	0.1565	0.42	0.0657	1	5	0.0001	0.00	0.0000	0	0
India	Devidhura Range, Uttarakhand, IN	IN4	0.1011	0.33	0.0334	1	4	0.1073	0.58	0.0626	0	7
India	Kheti, Uttarakhand, IN	IN5	0.2017	0.25	0.0504	1	3	0.4445	1.00	0.4445	2	12
India	Birgola, Uttarakhand, IN	IN6	0.1352	0.33	0.0446	1	4	0.1454	0.58	0.0848	0	7
India	Dharkot, Uttarakhand, IN	IN7	0.1403	0.17	0.0239	1	2	0.4967	1.00	0.4967	2	12
India	DPF Nanhani, Himachal Pradesh, IN	IN8	0.2034	0.25	0.0508	1	3	0.6364	1.00	0.6364	3	12
India	Bahru, Himachal Pradesh, IN	IN9	0.0996	0.33	0.0329	1	4	0.3148	0.75	0.2361	1	9
India	Chamba HP, IN	IN10	0.0785	0.50	0.0393	1	6	0.3786	0.92	0.3471	2	11
India	Lad Bharol HP, IN	IN11	0.0996	0.33	0.0329	1	4	0.3148	0.75	0.2361	1	9
India	Latti, Jammu and Kashmir, IN	IN12	0.0924	0.25	0.0231	1	3	0.2198	0.58	0.1282	1	7
India	Nar Sher Ali Khan, Jammu and Kashmir, IN	IN13	0.0457	0.25	0.0114	1	3	0.3483	0.75	0.2613	1	9
India	Bandipora, Jammu and Kashmir, IN	IN14	0.0168	0.25	0.0042	0	3	0.5941	1.00	0.5941	3	12
India	Shimla, Himachal Pradesh, IN	IN15	0.1720	0.17	0.0292	1	2	0.6135	1.00	0.6135	3	12

Region and Country	Location	Code	*Pgt* MINF (B)	Fav-month (F)	Favourable index (B x F)	Risk score	Month	Barberry NHTT (D)	Fav-month (F)	Favourable growth index (D x F)	Suitabilty score	Months
India	Rupal, Jammu and Kashmir, IN	IN16	0.0007	0.08	0.0001	0	1	0.5487	0.92	0.5030	3	11
India	Sankoo, Jammu and Kashmir, IN	IN17	0	0.00	0.0000	0	0	0.5509	0.83	0.4590	2	10
India	Magh Batna, Jammu and Kashmir, IN	IN18	0.0309	0.33	0.0102	1	4	0.6180	1.00	0.6180	3	12
India	Khawaspur, Punjab, IN	IN19	0.0472	0.25	0.0118	1	3	0.0818	0.42	0.0341	0	5
India	Dharm Manela, Himachal Pradesh, IN	IN20	0.0759	0.08	0.0061	0	1	0.6039	0.92	0.5536	3	11
India	Jamaga, Karnataka, IN	IN21	0.2636	0.33	0.0870	1	4	0.0052	0.33	0.0017	0	4
India	Waynad, Kerala, IN	IN22	0.4274	0.42	0.1795	2	5	0.0053	0.33	0.0018	0	4
India	Thanjavur, Tamil Nadu, IN	IN23	0.0358	0.25	0.0089	1	3	0.0002	0.00	0.0000	0	0
India	Mendpal, Chhattisgarh, IN	IN24	0.1618	0.25	0.0404	1	3	0.0196	0.42	0.0082	0	5
India	Basnara, Chhattisgarh, IN	IN25	0.0989	0.25	0.0247	1	3	0.0440	0.50	0.0220	0	6
India	Adiwas, Rajasthan, IN	IN26	0.0079	0.08	0.0006	0	1	0.0293	0.42	0.0122	0	5
India	Khesari Gidha, Uttar Pradesh, IN	IN27	0.0526	0.17	0.0089	1	2	0.0644	0.42	0.0269	0	5
India	Nandhaur Range, Uttarakhand, IN	IN28	0.1011	0.33	0.0334	1	4	0.1073	0.58	0.0626	0	7
India	Deema Range, Uttarakhand, IN	IN29	0.0872	0.33	0.0288	1	4	0.1116	0.58	0.0651	0	7
India	Bahru, Himachal Pradesh, IN	IN30	0.0996	0.33	0.0329	1	4	0.3148	0.75	0.2361	1	9
India	Bahl, Himachal Pradesh, IN	IN31	0.0768	0.25	0.0192	1	3	0.1429	0.58	0.0833	0	7
India	Golaghat, Assam, IN	IN32	0.0714	0.58	0.0414	1	7	0.0393	0.42	0.0164	0	5
India	Mokokchung, Nagaland, IN	IN33	0.0398	0.58	0.0231	1	7	0.0576	0.58	0.0336	0	7
India	Nagrasari, Rajasthan, IN	IN34	0	0.00	0.0000	0	0	0.0755	0.42	0.0315	0	5
India	Jind, Haryana, IN	IN35	0	0.00	0.0000	0	0	0.0955	0.42	0.0398	0	5
India	Thirpali Chhoti, Rajasthan, IN	IN36	0	0.00	0.0000	0	0	0.0768	0.42	0.0320	0	5
India	Roranwala Urf Tarewali, Punjab	IN37	0	0.00	0.0000	0	0	0.0930	0.42	0.0388	0	5
India	Gulbarga, Karnataka, IN	IN38	0.0578	0.25	0.0144	1	3	0.0070	0.33	0.0023	0	4
India	Poletipalle, Andhra Pradesh, IN	IN39	0.0098	0.33	0.0032	0	4	0.0044	0.33	0.0015	0	4
India	Marma, Maharashtra, IN	IN40	0	0.00	0.0000	0	0	0.0159	0.42	0.0066	0	5
India	Kaimur, Bihar, IN	IN41	0	0.00	0.0000	0	0	0.0493	0.50	0.0246	0	6
India	Karbi Anglog, Assam, IN	IN42	0.0501	0.58	0.0291	1	7	0.0328	0.42	0.0137	0	5
India	Champai, Mizoram, IN	IN43	0.0284	0.33	0.0094	1	4	0.0576	0.58	0.0336	0	7
India	Serchhip, Mizoram, IN	IN44	0.1212	0.42	0.0509	1	5	0.0364	0.42	0.0152	0	5

Region and Country	Location	Code	*Pgt* MINF (B)	*Pgt* Fav-month (F)	*Pgt* Favourable index (B x F)	*Pgt* Risk score	*Pgt* Month	Barberry NHTT (D)	Barberry Fav-month (F)	Barberry Favourable growth index (D x F)	Barberry Suitabilty score	Barberry Months
India	Baljata, J&K, IN	IN45	0.0717	0.17	0.0122	1	2	0.1267	0.58	0.0739	0	7
Mongolia	Unnamed, Lamaiin Huryee, MN	MN1	0.0031	0.08	0.0002	0	1	0.3531	1.00	0.3531	2	12
Mongolia	Unnamed, Khurmen, MN	MN2	0.0033	0.08	0.0003	0	1	0.3262	1.00	0.3262	2	12
Mongolia	Unnamed, Shinejinst, MN	MN3	0.0023	0.25	0.0006	0	3	0.3230	1.00	0.3230	2	12
Mongolia	Unnamed, Munkhkhairkhan, MN	MN4	0.0005	0.08	0.0000	0	1	0.4476	0.75	0.3357	2	9
Mongolia	Unnamed, Tsagaankhairkhan, MN	MN5	0.0186	0.25	0.0046	0	3	0.3573	0.83	0.2978	1	10
Mongolia	Unnamed, Orkhon, MN	MN6	0.0221	0.17	0.0038	0	2	0.3732	0.83	0.3109	2	10
Mongolia	Unnamed, Dashbalbar, MN	MN7	0.0091	0.25	0.0023	0	3	0.3200	0.83	0.2667	1	10
Mongolia	Unnamed, Matad, MN	MN8	0.0014	0.17	0.0002	0	2	0.3086	0.83	0.2571	1	10
Mongolia	Unnamed, Luus, MN	MN9	0.0037	0.08	0.0003	0	1	0.3349	0.83	0.2791	1	10
Mongolia	Unnamed, Khuvsgul, MN	MN10	0.0055	0.17	0.0009	0	2	0.3158	1.00	0.3158	2	12
Myanmar	Sagaing Region 1, Mym	MYN1	0	0.00	0.0000	0	0	0.0249	0.42	0.0104	0	5
Myanmar	Kyaukme, Mym	MYN2	0.0016	0.17	0.0003	0	2	0.0511	0.50	0.0256	0	6
Myanmar	Mindat, Mym	MYN3	0.0008	0.08	0.0001	0	1	0.0360	0.42	0.0150	0	5
Myanmar	Kyaukpyu, Mym	MYN4	0	0.00	0.0000	0	0	0.0066	0.25	0.0017	0	3
Myanmar	Rakhine, Mym	MYN5	0	0.00	0.0000	0	0	0.0004	0.00	0.0000	0	0
Myanmar	Putao 1, Mym	MYN6	0.0356	0.42	0.0150	1	5	0.1146	0.58	0.0669	0	7
Myanmar	Bhamo, Mym	MYN7	0.0049	0.25	0.0012	0	3	0.0270	0.42	0.0113	0	5
Myanmar	Sagaing Region 2, Mym	MYN8	0	0.00	0.0000	0	0	0.0080	0.25	0.0020	0	3
Nepal	Guthichaur, NP	NP1	0.2230	0.25	0.0557	1	3	0.7477	1.00	0.7477	4	12
Nepal	Namarjung, NP	NP2	0.3787	0.25	0.0947	1	3	0.5182	1.00	0.5182	3	12
Nepal	Maipokhari, NP	NP3	0.3290	0.25	0.0822	1	3	0.3880	1.00	0.3880	2	12
Nepal	Seti, NP	NP4	0.1350	0.25	0.0338	1	3	0.3754	1.00	0.3754	2	12
Nepal	Phopli 1, NP	NP5	0.1809	0.25	0.0452	1	3	0.2569	0.83	0.2140	1	10
Nepal	Phopli 2, NP	NP6	0.1352	0.25	0.0338	1	3	0.1454	0.58	0.0848	0	7
Nepal	Kailali, NP	NP7	0.0550	0.25	0.0138	1	3	0.0668	0.50	0.0334	0	6
Nepal	Bheri, NP	NP8	0.0503	0.25	0.0126	1	3	0.0625	0.50	0.0313	0	6
Nepal	Chitwan, NP	NP9	0.1964	0.25	0.0491	1	3	0.1178	0.58	0.0687	0	7
Nepal	Firfire, NP	NP10	0.3174	0.25	0.0793	1	3	0.4403	1.00	0.4403	2	12

			Pgt					Barberry				
Region and Country	Location	Code	MINF (B)	Fav-month (F)	Favourable index (B x F)	Risk score	Month	NHTT (D)	Fav-month (F)	Favourable growth index (D x F)	Suitabilty score	Months
Nepal	Huktumba, NP	NP11	0.1017	0.25	0.0254	1	3	0.0002	0.00	0.0000	0	0
Nepal	Khalanga, NP	NP12	0.0688	0.25	0.0172	1	3	0.0625	0.50	0.0313	0	6
Nepal	Tilepata, NP	NP13	0.2327	0.25	0.0582	1	3	0.5494	1.00	0.5494	3	12
Nepal	Khalanga NP	NP14	0.0503	0.25	0.0126	1	3	0.1178	0.58	0.0687	0	7
Nepal	Malatikot NP	NP15	0.1678	0.25	0.0419	1	3	0.5182	0.00	0.1296	3	12
Pakistan	Baricot, Khyber Pakhtunkhwa, PK	PK1	0.0442	0.50	0.0221	1	6	0.4048	0.75	0.3036	2	9
Pakistan	Abbottabad, Khyber Pakhtunkhwa, PK	PK2	0.0541	0.50	0.0271	1	6	0.3832	0.75	0.2874	1	9
Pakistan	Jand, Punjab, PK	PK3	0.0729	0.17	0.0124	1	2	0.1586	0.58	0.0925	0	7
Pakistan	Darsmand, Khyber Pakhtunkhwa, PK	PK4	0.0858	0.25	0.0214	1	3	0.2424	0.58	0.1414	1	7
Pakistan	Wana 1, Waziristan, PK	PK5	0.0314	0.25	0.0078	0	3	0.1905	0.58	0.1111	1	7
Pakistan	Qila Saifullah, Balochistan, PK	PK6	0	0.00	0.0000	0	0	0.4055	0.92	0.3717	2	11
Pakistan	Wana 2, Waziristan, PK	PK7	0.0189	0.25	0.0047	0	3	0.3253	0.75	0.2440	1	9
Pakistan	Awaran 1, Balochistan, PK	PK8	0	0.00	0.0000	0	0	0.0330	0.42	0.0137	0	5
Pakistan	Khuzdar, Balochistan, PK	PK9	0.0018	0.08	0.0001	0	1	0.2397	0.67	0.1598	1	8
Pakistan	Sangan, Balochistan, PK	PK10	0.0154	0.17	0.0026	0	2	0.1701	0.58	0.0992	0	7
Pakistan	Kalkot, Khyber Pakhtunkhwa, PK	PK11	0.0401	0.25	0.0100	1	3	0.5797	1.00	0.5797	3	12
Pakistan	Awaran 2, Balochistan, PK	PK12	0	0.00	0.0000	0	0	0.0821	0.42	0.0342	0	5
Pakistan	Mastuj, Khyber Pakhtunkhwa, PK	PK13	0	0.00	0.0000	0	0	0.5835	0.92	0.5349	3	11
Pakistan	Hunza Nagar, PK	PK14	0	0.00	0.0000	0	0	0.4345	0.58	0.2534	1	7
Pakistan	Alai, Khyber Pakhtunkhwa, PK	PK15	0.0214	0.33	0.0071	0	4	0.5449	1.00	0.5449	3	12
Pakistan	Bara, Khyber Agency, PK	PK16	0.0547	0.33	0.0181	1	4	0.3645	0.75	0.2734	1	9
	**Proportion of sites with infection index**	**0.756**										
**Central Asia (195)**									0.00			
Afghanistan	Khas Uruzgan, AFG	AFG1	0.0023	0.08	0.0002	0	1	0.5104	1.00	0.5104	3	12
Afghanistan	Berana, Panjab, AFG	AFG2	0	0.00	0.0000	0	0	0.5827	1.00	0.5827	3	12
Afghanistan	Kohistan, AFG	AFG3	0	0.00	0.0000	0	0	0.5798	1.00	0.5798	3	12
Afghanistan	Balkh, AFG	AFG4	0.0058	0.25	0.0015	0	3	0.4643	1.00	0.4643	2	12
Afghanistan	Warsaj, AFG	AFG5	0.0051	0.08	0.0004	0	1	0.8114	1.00	0.8114	4	12

			Pgt					Barberry				
Region and Country	Location	Code	MINF (B)	Fav-month (F)	Favourable index (B x F)	Risk score	Month	NHTT (D)	Fav-month (F)	Favourable growth index (D x F)	Suitabilty score	Months
Afghanistan	Alingar 1, AFG	AFG6	0.0040	0.17	0.0007	0	2	0.4667	0.92	0.4278	2	11
Afghanistan	Pur Chaman, AFG	AFG7	0.0009	0.08	0.0001	0	1	0.4720	0.92	0.4327	2	11
Afghanistan	Alingar 2, AFG	AFG8	0.0010	0.08	0.0001	0	1	0.5249	1.00	0.5249	3	12
Afghanistan	Sabari, AFG	AFG9	0	0.00	0.0000	0	0	0.4878	1.00	0.4878	2	12
Afghanistan	Bargi Matal, AFG	AFG10	0	0.00	0.0000	0	0	0.5669	0.92	0.5197	3	11
Afghanistan	Panjwai, AFG	AFG11	0.0025	0.08	0.0002	0	1	0.3004	0.75	0.2253	1	9
Afghanistan	Taywara, AFG	AFG12	0.0004	0.08	0.0000	0	1	0.5588	1.00	0.5588	3	12
Afghanistan	Sayyad, AFG	AFG13	0.0032	0.25	0.0008	0	3	0.4240	0.92	0.3887	2	11
Afghanistan	Bangi, AFG	AFG14	0.0265	0.42	0.0111	1	5	0.4844	1.00	0.4844	2	12
Afghanistan	Zinda Jan, AFG	AFG15	0.0005	0.08	0.0000	0	1	0.4134	0.92	0.3789	2	11
Afghanistan	Khamyab, AFG	AFG16	0.0145	0.17	0.0025	0	2	0.3646	0.75	0.2735	1	9
Afghanistan	Baghran, AFG	AFG17	0	0.00	0.0000	0	0	0.5241	1.00	0.5241	3	12
Afghanistan	Chihil Baghtoe Pashi, AFG	AFG18	0	0.00	0.0000	0	0	0.5679	1.00	0.5679	3	12
Afghanistan	Saghar, AFG	AFG19	0	0.00	0.0000	0	0	0.5670	1.00	0.5670	3	12
Afghanistan	Khidir, AFG	AFG20	0	0.00	0.0000	0	0	0.5674	1.00	0.5674	3	12
Azerbaijan	Alekseyevka, Khasmaz, AZ	AZ1	0.6765	0.75	0.5074	3	9	0.6180	0.83	0.5150	3	10
Azerbaijan	Derk, Quba, AZ	AZ2	0.0373	0.33	0.0123	1	4	0.5559	1.00	0.5559	3	12
Azerbaijan	Qaradagh, Baku, AZ	AZ3	0.1554	0.67	0.1041	2	8	0.5447	0.75	0.4085	2	9
Azerbaijan	Burkandul, Lerik, AZ	AZ4	0.2766	0.83	0.2295	2	10	0.5183	0.83	0.4319	2	10
Azerbaijan	Hasanabad, Neftchala, AZ	AZ5	0.9998	0.83	0.8298	4	10	0.4538	0.67	0.3026	2	8
Azerbaijan	Kurdchu, Hajiqabul, AZ	AZ6	0.4129	0.83	0.3427	3	10	0.2879	0.75	0.2159	1	9
Azerbaijan	Malikumudlu, Ujar, AZ	AZ7	0.0344	0.33	0.0113	1	4	0.4628	0.75	0.3471	2	9
Azerbaijan	Nagorno-Karabakh, AZ	AZ8	0.0462	0.42	0.0194	1	5	0.5246	0.92	0.4809	2	11
Azerbaijan	Alxanli, Fizuli, AZ	AZ9	0.0444	0.33	0.0147	1	4	0.4776	0.83	0.3979	2	10
Azerbaijan	Sarica, Shaki, AZ	AZ10	0.0477	0.33	0.0157	1	4	0.5075	0.83	0.4229	2	10
Azerbaijan	Qinli, Agstafa, AZ	AZ11	0.0774	0.42	0.0325	1	5	0.5170	0.83	0.4308	2	10
Azerbaijan	Damchili, Goygol, AZ	AZ12	0.0518	0.50	0.0259	1	6	0.5357	1.00	0.5357	3	12
Azerbaijan	Istisu, Nagorno-Karabakh, AZ	AZ13	0.0173	0.42	0.0073	0	5	0.5323	1.00	0.5323	3	12
Azerbaijan	Dizavar, Khizi, AZ	AZ14	0.1790	0.75	0.1343	2	9	0.5712	0.83	0.4760	2	10

			Pgt					Barberry				
Region and Country	Location	Code	MINF (B)	Fav-month (F)	Favourable index (B x F)	Risk score	Month	NHTT (D)	Fav-month (F)	Favourable growth index (D x F)	Suitabilty score	Months
Azerbaijan	Khinaliq, Qusar, AZ	AZ15	0.0373	0.33	0.0123	1	4	0.5559	1.00	0.5559	3	12
Azerbaijan	Solguja, Oghuz, AZ	AZ16	0.0266	0.33	0.0088	1	4	0.5508	1.00	0.5508	3	12
Iran	Ahar, East Azerbaijan, IRN	IRN1	0.0151	0.33	0.0050	0	4	0.5355	1.00	0.5355	3	12
Iran	Tang-e-Panj, Khuzestan, IRN	IRN2	0.0402	0.33	0.0133	1	4	0.3282	0.67	0.2188	1	8
Iran	Aghowz Darban, Mazandaran, IRN	IRN3	0.0338	0.42	0.0142	1	5	0.5329	1.00	0.5329	3	12
Iran	Hakan, Fars, IRN	IRN4	0	0.00	0.0000	0	0	0.2779	0.67	0.1853	1	8
Iran	Far-Ashian, North Khorasan, IRN	IRN5	0.0040	0.08	0.0003	0	1	0.4581	0.92	0.4200	2	11
Iran	Ferdows, South Khorasan, IRN	IRN6	0.0708	0.42	0.0297	1	5	0.3684	0.75	0.2763	1	9
Iran	Gale Koor, Lorestan, IRN	IRN7	0	0.00	0.0000	0	0	0.0205	0.25	0.0051	0	3
Iran	Tabriz, East Azerbaijan, IRN	IRN8	0.0163	0.25	0.0041	0	3	0.5223	1.00	0.5223	3	12
Iran	Basak, Gilan, IRN	IRN9	0.1419	0.42	0.0596	1	5	0.5669	1.00	0.5669	3	12
Iran	Arab Lang, Kurdistan, IRN	IRN10	0.0015	0.08	0.0001	0	1	0.5269	1.00	0.5269	3	12
Iran	West Azerbaijan, IRN	IRN11	0.0145	0.25	0.0036	0	3	0.5338	1.00	0.5338	3	12
Iran	Bilesavar, Ardabil, IRN	IRN12	0.0515	0.33	0.0170	1	4	0.4914	0.92	0.4505	2	11
Iran	Kharfegil, Gilan, IRN	IRN13	0.1419	0.42	0.0596	1	5	0.5669	1.00	0.5669	3	12
Iran	Charoymaq, East Azerbaijan, IRN	IRN14	0.0034	0.08	0.0003	0	1	0.5349	1.00	0.5349	3	12
Iran	Kafteroud, Gilan, IRN	IRN15	0.4819	0.83	0.4000	3	10	0.4461	0.83	0.3718	2	10
Iran	Gadel, Zanjan, IRN	IRN16	0.0053	0.17	0.0009	0	2	0.5226	1.00	0.5226	3	12
Iran	Roudbar Alamout, Qazvin, IRN	IRN17	0.0782	0.42	0.0329	1	5	0.5496	1.00	0.5496	3	12
Iran	Tamisan, Tehran, IRN	IRN18	0.0089	0.25	0.0022	0	3	0.5115	1.00	0.5115	3	12
Iran	Qarehtappeh, Mazandaran, IRN	IRN19	0.3946	0.75	0.2960	2	9	0.3327	0.67	0.2218	1	8
Iran	Narlidaq, Golestan, IRN	IRN20	0.0859	0.33	0.0284	1	4	0.3547	0.67	0.2365	1	8
Iran	Shirvan, North Khorasan, IRN	IRN21	0.0006	0.08	0.0000	0	1	0.4849	1.00	0.4849	2	12
Iran	Mozduran, Razavi Khorasan, IRN	IRN22	0.0101	0.17	0.0017	0	2	0.3961	0.75	0.2970	1	9
Iran	Shahrud, Semnan, IRN	IRN23	0	0.00	0.0000	0	0	0.4057	0.75	0.3043	2	9
Iran	Abadan, Khuzestan, IRN	IRN24	0.0326	0.17	0.0055	0	2	0.0911	0.50	0.0456	0	6
Iran	Anjiru, Bushehr, IRN	IRN25	0.1725	0.50	0.0863	1	6	0.0307	0.42	0.0128	0	5
Iran	Abdan, Bushehr, IRN	IRN26	0.0914	0.50	0.0457	1	6	0.0119	0.33	0.0040	0	4
Iran	Garisheh, Hormozgan, IRN	IRN27	0.0094	0.33	0.0031	0	4	0.0098	0.33	0.0033	0	4

Region and Country	Location	Code	*Pgt* MINF (B)	Fav-month (F)	Favourable index (B x F)	Risk score	Month	Barberry NHTT (D)	Fav-month (F)	Favourable growth index (D x F)	Suitabilty score	Months
Iran	Tifakan, Hormozgan, IRN	IRN28	0.0010	0.08	0.0001	0	1	0.0879	0.50	0.0440	0	6
Iran	Kasi, Sistan and Balochistan, IRN	IRN29	0.0021	0.17	0.0004	0	2	0.0144	0.33	0.0048	0	4
Iran	Khash, Sistan and Balochistan, IRN	IRN30	0	0.00	0.0000	0	0	0.2331	0.67	0.1554	1	8
Iran	Zarand, Kerman, IRN	IRN31	0	0.00	0.0000	0	0	0.3813	0.92	0.3495	2	11
Iran	Reza Abad, Fars, IRN	IRN32	0	0.00	0.0000	0	0	0.4779	1.00	0.4779	2	12
Iran	Sirusabad, Yazd, IRN	IRN33	0	0.00	0.0000	0	0	0.3507	0.75	0.2630	1	9
Iran	Kashan County, Isfahan, IRN	IRN34	0.0010	0.08	0.0001	0	1	0.4711	0.92	0.4319	2	11
Iran	Jera, Hamadan, IRN	IRN35	0.0040	0.17	0.0007	0	2	0.5184	1.00	0.5184	3	12
Iran	Cheshme, Markazi, IRN	IRN36	0.0010	0.08	0.0001	0	1	0.4737	0.92	0.4342	2	11
Iran	Mahdishahr, Semnan, IRN	IRN37	0.0022	0.08	0.0002	0	1	0.4870	1.00	0.4870	2	12
Iran	Shahrud 2, Semnan, IRN	IRN38	0	0.00	0.0000	0	0	0.3724	0.75	0.2793	1	9
Iran	Dehrud, Fars, IRN	IRN39	0.0021	0.08	0.0002	0	1	0.2632	0.67	0.1755	1	8
Iran	Urmia, West Azerbaijan, IRN	IRN40	0.0245	0.25	0.0061	0	3	0.5339	1.00	0.5339	3	12
Iran	Chalus, Mazandaran, IRN	IRN41	0.5133	0.83	0.4261	3	10	0.5125	0.83	0.4271	2	10
Iran	Pourva, Mazandaran, IRN	IRN42	0.3946	0.75	0.2960	2	9	0.3327	0.67	0.2218	1	8
Iran	Lulak-E Lebur, Mazandaran, IRN	IRN43	0.0478	0.42	0.0201	1	5	0.4698	1.00	0.4698	2	12
Iran	Pust Kola, Mazandaran, IRN	IRN44	0.0708	0.42	0.0297	1	5	0.4911	1.00	0.4911	2	12
Iran	Manuchehrkela, Mazandaran, IRN	IRN45	0.9091	0.83	0.7546	4	10	0.4198	0.67	0.2799	1	8
Iran	Shalma Shaft, Gilan, IRN	IRN46	0.2351	0.75	0.1764	2	9	0.4991	0.83	0.4159	2	10
Iran	Askabon, Gilan, IRN	IRN47	0.0574	0.33	0.0189	1	4	0.5166	1.00	0.5166	3	12
Iran	GolRoudbar, Gilan, IRN	IRN48	0.4819	0.83	0.4000	3	10	0.4461	0.83	0.3718	2	10
Iran	Harandan, Gilan, IRN	IRN49	0.1944	0.50	0.0972	1	6	0.5645	1.00	0.5645	3	12
Iran	Rudakanar, Masally, AZ	IRN50	0.2766	0.83	0.2295	2	10	0.5183	0.83	0.4319	2	10
Iran	Shaft, Gilan, IRN	IRN51	0.2351	0.75	0.1764	2	9	0.4991	0.83	0.4159	2	10
Iran	Shiveh Berow, West Azerbaijan, IRN	IRN52	0.0236	0.17	0.0040	0	2	0.5390	1.00	0.5390	3	12
Iran	Akhond Mahalleh, Mazandaran, IRN	IRN53	0.2286	0.75	0.1715	2	9	0.5683	1.00	0.5683	3	12
Iran	Chanasak, Qazvin, IRN	IRN54	0.0128	0.25	0.0032	0	3	0.5099	1.00	0.5099	3	12
Iran	Kuchek Khortum, Golestan, IRN	IRN55	0.0789	0.33	0.0260	1	4	0.3606	0.67	0.2404	1	8
Iran	Viva, Golestan, IRN	IRN56	0.2123	0.75	0.1592	2	9	0.3987	0.67	0.2658	1	8

Region and Country	Location	Code	*Pgt* MINF (B)	*Pgt* Fav-month (F)	*Pgt* Favourable index (B x F)	*Pgt* Risk score	*Pgt* Month	Barberry NHTT (D)	Barberry Fav-month (F)	Barberry Favourable growth index (D x F)	Barberry Suitabilty score	Barberry Months
Iran	Paeen Ahmad, Mazandaran, IRN	IRN57	0.6008	0.75	0.4506	3	9	0.3073	0.67	0.2049	1	8
Iran	Zirab, Mazandaran, IRN	IRN58	0	0.08	0.0000	0	1	0.4911	1.00	0.4911	2	12
Kazakhstan	Aksu, Karakiya, KZ	KZ1	0.0004	0.08	0.0000	0	1	0.4804	0.83	0.4003	2	10
Kazakhstan	Karakum, Tupkaragan, KZ	KZ2	0.1232	0.58	0.0715	1	7	0.5753	0.75	0.4315	2	9
Kazakhstan	Unnamed 2, Karakiya, KZ	KZ3	0.0058	0.17	0.0010	0	2	0.3954	0.92	0.3625	2	11
Kazakhstan	Unnamed, Karakum, Tupkaragan, KZ	KZ4	0.1286	0.33	0.0425	1	4	0.4266	0.75	0.3199	2	9
Kazakhstan	Unnamed, Isatay, KZ	KZ5	0.0271	0.33	0.0089	1	4	0.3439	0.92	0.3153	2	11
Kazakhstan	Kulandy, Aral, KZ	KZ6	0.0728	0.33	0.0240	1	4	0.3000	0.92	0.2750	1	11
Kazakhstan	Bis-Bulyuk, Makat, KZ	KZ7	0.0357	0.25	0.0089	1	3	0.3267	0.92	0.2995	1	11
Kazakhstan	Kimer-Sayek-Kul', Syrym, KZ	KZ8	0.0621	0.25	0.0155	1	3	0.3075	1.00	0.3075	2	12
Kazakhstan	Akhol, Ayteke Bi, KZ	KZ9	0.0574	0.33	0.0190	1	4	0.2831	0.83	0.2359	1	10
Kazakhstan	Aiyrtau, Airtau, KZ	KZ10	0.1374	0.42	0.0577	1	5	0.3336	0.75	0.2502	1	9
Kazakhstan	L'vovka, Kashyr, KZ	KZ11	0.1239	0.33	0.0409	1	4	0.3228	0.75	0.2421	1	9
Kazakhstan	Sartymbet, Ulan, KZ	KZ12	0.0536	0.33	0.0177	1	4	0.3285	0.83	0.2737	1	10
Kazakhstan	Unnamed, Bukhar-Zhyrau, KZ	KZ13	0.0267	0.42	0.0112	1	5	0.0012	0.00	0.0000	0	0
Kazakhstan	Unnamed, Nura, KZ	KZ14	0.0598	0.33	0.0197	1	4	0.3177	0.83	0.2647	1	10
Kazakhstan	Unnamed, South Kazakhstan, KZ	KZ15	0.0211	0.25	0.0053	0	3	0.3927	0.83	0.3273	2	10
Kazakhstan	Unnamed, Bayganin, KZ	KZ16	0.0756	0.33	0.0250	1	4	0.2891	0.83	0.2409	1	10
Kazakhstan	Goloschchekin, Atyrau, KZ	KZ17	0.0472	0.42	0.0198	1	5	0.3969	0.92	0.3639	2	11
Kazakhstan	Unnamed, Atyrau, KZ	KZ18	0.0483	0.33	0.0159	1	4	0.3216	0.92	0.2948	1	11
Kyrgyzstan	Issyk-Kul, KGN	KGN1	0.0258	0.17	0.0044	0	2	0.3689	0.92	0.3382	2	11
Kyrgyzstan	Temir-Kanat, Tong, KGN	KGN2	0.0643	0.42	0.0270	1	5	0.5107	1.00	0.5107	3	12
Kyrgyzstan	Ak-Su 1, KGN	KGN3	0	0.00	0.0000	0	0	0.5270	1.00	0.5270	3	12
Kyrgyzstan	Naryn, At-Bashi, KGN	KGN4	0	0.00	0.0000	0	0	0.5739	0.83	0.4782	2	10
Kyrgyzstan	Lyangar, Kadamzhai, KGN	KGN5	0	0.00	0.0000	0	0	0.5594	0.92	0.5128	3	11
Kyrgyzstan	Uzak, Aravan, KGN	KGN6	0.0073	0.17	0.0012	0	2	0.5953	1.00	0.5953	3	12
Kyrgyzstan	Terek, Ak-Talaa, KGN	KGN7	0.0058	0.08	0.0005	0	1	0.4737	1.00	0.4737	2	12
Kyrgyzstan	Karabashat 1, Aksy, KGN	KGN8	0.0004	0.08	0.0000	0	1	0.5394	1.00	0.5394	3	12
Kyrgyzstan	Ak-Su 2, KGN	KGN9	0	0.00	0.0000	0	0	0.5537	1.00	0.5537	3	12

Region and Country	Location	Code	*Pgt* MINF (B)	Fav-month (F)	Favourable index (B x F)	Risk score	Month	Barberry NHTT (D)	Fav-month (F)	Favourable growth index (D x F)	Suitabilty score	Months
Kyrgyzstan	Konduk, Kara-Kulja, KGN	KGN10	0.0036	0.08	0.0003	0	1	0.4939	1.00	0.4939	2	12
Kyrgyzstan	Karabashat 2, Aksy, KGN	KGN11	0.0004	0.08	0.0000	0	1	0.5582	0.75	0.4186	2	9
Kyrgyzstan	Panfilov, KGN	KGN12	0.0048	0.08	0.0004	0	1	0.5708	1.00	0.5708	3	12
Kyrgyzstan	Chot-Nura, Naryn, KGN	KGN13	0.0048	0.08	0.0004	0	1	0.0527	0.75	0.0395	0	9
Kyrgyzstan	Issyk-kul region, KGN	KGN14	0.0068	0.17	0.0012	0	2	0.5451	0.92	0.4997	2	11
Russia	Rebrikhinsky, Altai Krai, RS	RS1	0.1130	0.33	0.0373	1	4	0.3329	0.75	0.2497	1	9
Russia	Miguty, Krasnodor Krai, RS	RS2	0.0653	0.42	0.0274	1	5	0.5130	0.92	0.4702	2	11
Russia	Indyuk, Krasnodor Krai, RS	RS3	0.3754	0.75	0.2815	2	9	0.5911	1.00	0.5911	3	12
Russia	Rassvet, Volgograd Oblast, RS	RS4	0.0542	0.33	0.0179	1	4	0.4279	0.92	0.3922	2	11
Russia	Tyurinsky, Volgograd Oblast, RS	RS5	0.0545	0.33	0.0180	1	4	0.3541	1.00	0.3541	2	12
Russia	Sadovyy, Volgograd Oblast, RS	RS6	0.0537	0.25	0.0134	1	3	0.3546	1.00	0.3546	2	12
Russia	Grabovskiy, Rostov Oblast, RS	RS7	0.0517	0.50	0.0258	1	6	0.4536	0.92	0.4158	2	11
Russia	Nogaysky, Dagetsan Republic, RS	RS8	0.0388	0.33	0.0128	1	4	0.5127	0.83	0.4272	2	10
Russia	Verkh Karanay, Dagestan Reoublic, RS	RS9	0.0564	0.33	0.0186	1	4	0.5398	1.00	0.5398	3	12
Russia	Novourupskiy, Krasnodar Krai, RS	RS10	0.0965	0.33	0.0319	1	4	0.5388	1.00	0.5388	3	12
Russia	Indyuk, Krasnodor Krai, RS	RS11	0.3754	0.75	0.2815	2	9	0.5911	1.00	0.5911	3	12
Russia	Kiziltashskiy Liman, Krasnodar Krai, RS	RS12	0.5798	0.75	0.4349	3	9	0.6095	0.83	0.5079	3	10
Russia	Oktyabr'skiy, Krasnodar Krai, RS	RS13	0.2365	0.50	0.1183	2	6	0.5733	0.83	0.4777	2	10
Russia	Sadovyy, Volgograd Oblast, RS	RS14	0.0377	0.25	0.0094	1	3	0.4324	1.00	0.4324	2	12
Russia	Grabovskiy, Rostov Oblast, RS	RS15	0.2135	0.33	0.0704	1	4	0.8407	1.00	0.8407	4	12
Russia	Nogaysky, Dagetsan Republic, RS	RS16	0.0356	0.25	0.0089	1	3	0.3345	0.58	0.1951	1	7
Russia	Verkh Karanay, Dagestan Reoublic, RS	RS17	0.1157	0.33	0.0382	1	4	0.3032	0.58	0.1769	1	7
Russia	Novourupskiy, Krasnodar Krai, RS	RS18	0.0753	0.33	0.0249	1	4	0.3301	0.75	0.2475	1	9
Russia	Goytkh, Krasnodar Krai, RS	RS19	0.2240	0.50	0.1120	2	6	0.3578	0.75	0.2683	1	9
Russia	Volodarsky, Astrakhan Oblast, RS	RS20	0.0939	0.42	0.0394	1	5	0.4406	0.83	0.3671	2	10
Russia	Kalmykia, RS	RS21	0.0404	0.33	0.0133	1	4	0.4202	0.92	0.3852	2	11
Russia	Grushevo, Astrakhan Oblast, RS	RS22	0.0470	0.42	0.0197	1	5	0.4335	0.92	0.3974	2	11
Russia	Khivsky, Dagestan, RS	RS23	0.1038	0.50	0.0519	1	6	0.5752	1.00	0.5752	3	12
Tajikistan	Ardzhanak, Hisor, TJ	TJ1	0.0068	0.17	0.0012	0	2	0.4922	1.00	0.4922	2	12

Region and Country	Location	Code	*Pgt* MINF (B)	Fav-month (F)	Favourable index (B x F)	Risk score	Month	Barberry NHTT (D)	Fav-month (F)	Favourable growth index (D x F)	Suitabilty score	Months
Tajikistan	Kaltashur, TJ	TJ2	0.0150	0.33	0.0049	0	4	0.3897	0.75	0.2923	1	9
Tajikistan	Arabkhana, Shahrtuz, TJ	TJ3	0.0185	0.33	0.0061	0	4	0.3669	0.75	0.2752	1	9
Tajikistan	Unnamed, Gorno-Badakhshan, TJ	TJ4	0	0.00	0.0000	0	0	0.4327	0.75	0.3246	2	9
Tajikistan	Khamirabot, Ghafurov, TJ	TJ5	0.0475	0.33	0.0157	1	4	0.4625	0.92	0.4240	2	11
Tajikistan	Karakendzha, Jirgatol, TJ	TJ6	0	0.00	0.0000	0	0	0.5594	0.83	0.4661	2	10
Tajikistan	Shahrinav, Shakhrinaw, TJ	TJ7	0.0012	0.08	0.0001	0	1	0.4937	1.00	0.4937	2	12
Tajikistan	Medynved, Roshtqala, TJ	TJ8	0	0.00	0.0000	0	0	0.5006	0.75	0.3754	2	9
Tajikistan	Vose, TJ	TJ9	0.0141	0.42	0.0059	0	5	0.4515	0.83	0.3762	2	10
Tajikistan	Shurpast, Varzov, TJ	TJ10	0.0009	0.08	0.0001	0	1	0.5377	1.00	0.5377	3	12
Tajikistan	Qurgonteppa, Sarband, TJ	TJ11	0.0218	0.33	0.0072	0	4	0.3703	0.75	0.2778	1	9
Tajikistan	Arzing, Tavildara, TJ	TJ12	0	0.00	0.0000	0	0	0.5838	0.92	0.5352	3	11
Tajikistan	Barchidev, Rushon, TJ	TJ13	0	0.00	0.0000	0	0	0.4111	0.67	0.2741	1	8
Turkmenistan	Karagez, Balkan, TKM	TKM1	0.0399	0.25	0.0100	1	3	0.4088	0.75	0.3066	2	9
Turkmenistan	Syul'men, Balkan, TKM	TKM2	0.0227	0.33	0.0075	0	4	0.4924	0.75	0.3693	2	9
Turkmenistan	Aydin, Balkan, TKM	TKM3	0.0158	0.17	0.0027	0	2	0.4268	0.75	0.3201	2	9
Turkmenistan	Ekerem, Balkan, TKM	TKM4	0.4045	0.83	0.3358	3	10	0.4196	0.67	0.2798	1	8
Turkmenistan	Bekibent, Balkan, TKM	TKM5	0.0399	0.25	0.0100	1	3	0.4088	0.75	0.3066	2	9
Turkmenistan	Kabyl, Balkan, TKM	TKM6	0.0227	0.33	0.0075	0	4	0.4924	0.75	0.3693	2	9
Turkmenistan	Unnamed 1, Balkan, TKM	TKM7	0	0.00	0.0000	0	0	0.4387	0.75	0.3290	2	9
Turkmenistan	Unnamed 2, Balkan, TKM	TKM8	0.0058	0.25	0.0015	0	3	0.4393	0.75	0.3295	2	9
Turkmenistan	Kaplankyr Reserve1, Dashoguz, TKM	TKM9	0.0054	0.17	0.0009	0	2	0.4109	0.75	0.3081	2	9
Turkmenistan	Kaplankyr Reserve 2, Dashoguz, TKM	TKM10	0.0145	0.25	0.0036	0	3	0.3992	0.75	0.2994	1	9
Turkmenistan	Shakhnamy, Dasoguz, TKM	TKM11	0.0043	0.17	0.0007	0	2	0.3852	0.75	0.2889	1	9
Turkmenistan	Unnamed 1, Mary, TKM	TKM12	0.0014	0.08	0.0001	0	1	0.3872	0.75	0.2904	1	9
Turkmenistan	Dervezekem, Mary, TKM	TKM13	0.0044	0.17	0.0008	0	2	0.3835	0.75	0.2876	1	9
Turkmenistan	Batash, Lebap, TKM	TKM14	0.0129	0.17	0.0022	0	2	0.3708	0.75	0.2781	1	9
Turkmenistan	Unnamed 2, Mary, TKM	TKM15	0.0140	0.25	0.0035	0	3	0.3984	0.75	0.2988	1	9
Turkmenistan	Unnamed , Ahal, TKM	TKM16	0.0140	0.25	0.0035	0	3	0.4111	0.75	0.3083	2	9
Turkmenistan	Serbar, Balkan, TKM	TKM17	0.0236	0.17	0.0040	0	2	0.4162	0.75	0.3121	2	9

			Pgt					Barberry				
Region and Country	Location	Code	MINF (B)	Fav-month (F)	Favourable index (B x F)	Risk score	Month	NHTT (D)	Fav-month (F)	Favourable growth index (D x F)	Suitabilty score	Months
Turkmenistan	Unnamed 3, Balkan, TKM	TKM18	0.0064	0.17	0.0011	0	2	0.4295	0.75	0.3221	2	9
Turkmenistan	Unnamed, Dashoguz, TKM	TKM19	0.0085	0.33	0.0028	0	4	0.4079	0.75	0.3059	2	9
Turkmenistan	Unnamed 4, Balkan, TKM	TKM20	0.0046	0.08	0.0004	0	1	0.4223	0.75	0.3167	2	9
Uzbekistan	Shahrisabz, Yakkabog, UZ	UZ1	0.0211	0.17	0.0036	0	2	0.4773	0.92	0.4375	2	11
Uzbekistan	Unnamed, Tamdy, UZ	UZ2	0.0233	0.08	0.0019	0	1	0.3867	0.92	0.3545	2	11
Uzbekistan	Unnamed 1, Muynak, UZ	UZ3	0.1262	0.33	0.0416	1	4	0.3529	0.75	0.2647	1	9
Uzbekistan	Unnamed 1, Kungrad, UZ	UZ4	0.0398	0.17	0.0068	0	2	0.3258	0.92	0.2986	1	11
Uzbekistan	Unnamed 2, Kungrad, UZ	UZ5	0.0108	0.08	0.0009	0	1	0.4040	0.75	0.3030	2	9
Uzbekistan	Vozrozhdeniya, UZ	UZ6	0.4618	0.67	0.3094	3	8	0.3267	0.75	0.2450	1	9
Uzbekistan	Unnamed 3, Kungrad, UZ	UZ7	0.0251	0.08	0.0020	0	1	0.3183	0.92	0.2918	1	11
Uzbekistan	Unnamed 4, Kungrad, UZ	UZ8	0.1169	0.33	0.0386	1	4	0.3137	0.75	0.2353	1	9
Uzbekistan	Unnamed 1, Karakiya, KZ	UZ9	0.0059	0.00	0.0000	0	0	0.4280	0.75	0.3210	2	9
Uzbekistan	Unnamed 2, Muynak, UZ	UZ10	0.4476	0.67	0.2999	2	8	0.3372	0.75	0.2529	1	9
Uzbekistan	Unnamed 3, Muynak, UZ	UZ11	0.1056	0.33	0.0349	1	4	0.3436	0.75	0.2577	1	9
Uzbekistan	Kungrad, UZ	UZ12	0.0092	0.17	0.0016	0	2	0.3562	0.92	0.3266	2	11
Uzbekistan	Ahangaran, UZ	UZ13	0.0577	0.33	0.0190	1	4	0.2601	0.00	0.3121	2	10
	**Proportion of sites with infection index**	**0.728**										
**Northwest Asia (59)**									0.00			
Armenia	Lernahovit, Gegharkunik, ARM	ARM1	0.0458	0.42	0.0192	1	5	0.5338	1.00	0.5338	3	12
Armenia	Norekart, Gegharkunik, ARM	ARM2	0.0402	0.42	0.0169	1	5	0.5526	1.00	0.5526	3	12
Armenia	Gandzakar, Tavush, ARM	ARM3	0.0525	0.42	0.0220	1	5	0.5531	1.00	0.5531	3	12
Armenia	Krasar, Shirak, ARM	ARM4	0.0426	0.33	0.0140	1	4	0.5393	1.00	0.5393	3	12
Armenia	Armavir, Armavir, ARM	ARM5	0.0165	0.25	0.0041	0	3	0.5164	1.00	0.5164	3	12
Armenia	Semyonovka, Gegharkunik, ARM	ARM6	0.0460	0.42	0.0193	1	5	0.5491	1.00	0.5491	3	12
Armenia	Daranak, Gegharkunik, ARM	ARM7	0.0402	0.42	0.0169	1	5	0.5526	1.00	0.5526	3	12
Armenia	Artsvanist, Gegharkunik, ARM	ARM8	0.0402	0.42	0.0169	1	5	0.5526	1.00	0.5526	3	12
Armenia	Tsovazard, Gegharkunik, ARM	ARM9	0.0458	0.42	0.0192	1	5	0.5338	1.00	0.5338	3	12
Armenia	Dilijan, Bovakar Ridge, ARM	ARM10	0.0460	0.42	0.0193	1	5	0.5491	1.00	0.5491	3	12

			Pgt					Barberry				
Region and Country	Location	Code	MINF (B)	Fav-month (F)	Favourable index (B x F)	Risk score	Month	NHTT (D)	Fav-month (F)	Favourable growth index (D x F)	Suitabilty score	Months
Georgia	Likhni, GRG	GRG1	0.3954	0.75	0.2966	2	9	0.1958	1.00	0.1958	1	12
Georgia	Lukhi, Samegrelo-Zemo Svaneti, GRG	GRG2	0.1749	0.50	0.0874	1	6	0.2460	1.00	0.2460	1	12
Georgia	Ergeta, Samegrelo-Zemo Svaneti, GRG	GRG3	0.8740	1.00	0.8740	4	12	0.0544	0.83	0.0454	0	10
Georgia	Zeda sameba, GRG	GRG4	0.6838	1.00	0.6838	3	12	0.1080	1.00	0.1080	1	12
Georgia	Kvaisi, South Ossetia, GRG	GRG5	0.0513	0.42	0.0216	1	5	0.2964	1.00	0.2964	1	12
Georgia	Rustavi, Kvemo Kartli, GRG	GRG6	0.0623	0.50	0.0312	1	6	0.1677	1.00	0.1677	1	12
Georgia	Telavi, Kakheti, GRG	GRG7	0.0577	0.42	0.0242	1	5	0.1885	1.00	0.1885	1	12
Georgia	Lahili, Samegrelo-Zemo Svaneti, GRG	GRG8	0.1269	0.50	0.0635	1	6	0.3244	1.00	0.3244	2	12
Georgia	Vabiskhevi, Borjomi nature Reserve, GRG	GRG9	0.0756	0.58	0.0438	1	7	0.2764	0.75	0.2073	1	9
Turkey	Ilyaslar, Bolu, TKY	TKY1	0.0353	0.33	0.0116	1	4	0.5885	1.00	0.5885	3	12
Turkey	Buyukcakirman, Erzincan, TKY	TKY2	0.0271	0.33	0.0089	1	4	0.5672	1.00	0.5672	3	12
Turkey	Karakoy, Denizli, TKY	TKY3	0.0130	0.17	0.0022	0	2	0.5004	0.92	0.4587	2	11
Turkey	Gercam Mahallesi, Denizli, TKY	TKY4	0.0382	0.25	0.0096	1	3	0.4779	0.83	0.3982	2	10
Turkey	Sagrak Koyu, Isparta, TKY	TKY5	0.0127	0.17	0.0022	0	2	0.5905	1.00	0.5905	3	12
Turkey	Cesmelisebil, Konya, TKY	TKY6	0.0058	0.17	0.0010	0	2	0.5453	1.00	0.5453	3	12
Turkey	Isikkaya Mahallesi, Adana, TKY	TKY7	0.0172	0.25	0.0043	0	3	0.5501	1.00	0.5501	3	12
Turkey	Kaymakli, Erzican, TKY	TKY8	0.0157	0.17	0.0027	0	2	0.5336	1.00	0.5336	3	12
Turkey	kale Koyu, Rize, TKY	TKY9	0.1722	0.58	0.0999	1	7	0.5668	1.00	0.5668	3	12
Turkey	Merkez mahallesi, Sinop, TKY	TKY10	0.2056	0.58	0.1192	2	7	0.5930	1.00	0.5930	3	12
Turkey	Sulek Koyu, Bartin, TKY	TKY11	0.1219	0.42	0.0512	1	5	0.5667	1.00	0.5667	3	12
Turkey	Sayalar Mahallesi, Istanbul, TKY	TKY12	0.6046	0.83	0.5018	3	10	0.5132	0.83	0.4277	2	10
Turkey	Camliova Mahallesi, Antalya, TKY	TKY13	0.8624	0.75	0.6468	3	9	0.1368	0.67	0.0912	0	8
Turkey	Agackoy Koyu, Canakkale, TKY	TKY14	0.1798	0.58	0.1043	2	7	0.4533	0.83	0.3778	2	10
Turkey	Dogla Mahallesi, Bursa, TKY	TKY15	0.3396	0.83	0.2819	2	10	0.4830	0.83	0.4025	2	10
Turkey	Kaleici Mahallesi, Istanbul, TKY	TKY16	0.6046	0.83	0.5018	3	10	0.5132	0.83	0.4277	2	10
Turkey	Camkoy Koyu, Canakkale, TKY	TKY17	0.2924	0.83	0.2427	2	10	0.4588	0.83	0.3824	2	10
Turkey	Musellim Koyu, Kirklareli, TKY	TKY18	0.0726	0.33	0.0240	1	4	0.4923	0.83	0.4103	2	10
Turkey	Canakca Mahllesi, Istanbul, TKY	TKY19	0.6046	0.83	0.5018	3	10	0.5132	0.83	0.4277	2	10
Turkey	Imamlar Mahallesi, Sakarya, TKY	TKY20	0.9057	0.92	0.8332	4	11	0.5138	0.83	0.4282	2	10

Region and Country	Location	Code	*Pgt* MINF (B)	*Pgt* Fav-month (F)	*Pgt* Favourable index (B x F)	*Pgt* Risk score	*Pgt* Month	Barberry NHTT (D)	Barberry Fav-month (F)	Barberry Favourable growth index (D x F)	Barberry Suitabilty score	Barberry Months
Turkey	Haydarlar Mahallesi, Ankara, TKY	TKY21	0.0155	0.25	0.0039	0	3	0.5708	1.00	0.5708	3	12
Turkey	Ezelter Koyu, Giresun, TKY	TKY22	0.1583	0.58	0.0918	1	7	0.6227	1.00	0.6227	3	12
Turkey	Ortaklar Koyu, Rize, TKY	TKY23	0.1722	0.58	0.0999	1	7	0.5668	1.00	0.5668	3	12
Turkey	Esmepinar Koyu, Erzican, TKY	TKY24	0.0029	0.17	0.0005	0	2	0.5251	1.00	0.5251	3	12
Turkey	Yolgecen, Artvin, TKY	TKY25	0.5354	0.83	0.4444	3	10	0.6126	1.00	0.6126	3	12
Turkey	Kaplanli Kyo, Afyonkarahisar, TKY	TKY26	0.0160	0.33	0.0053	0	4	0.5853	1.00	0.5853	3	12
Turkey	Kokulupinar Koyu, Agri, TKY	TKY27	0	0.00	0.0000	0	0	0.5154	1.00	0.5154	3	12
Turkey	Sekirhan, Diyarbakir, TKY	TKY28	0.0187	0.17	0.0032	0	2	0.4979	1.00	0.4979	2	12
Turkey	Bulduk Mahallesi, Konya, TKY	TKY29	0.0095	0.17	0.0016	0	2	0.5516	1.00	0.5516	3	12
Turkey	Yeni Mahallesi, Denizli, TKY	TKY30	0.0130	0.17	0.0022	0	2	0.5004	0.92	0.4587	2	11
Turkey	Ceceli Mahallesi, Kahramanmaras, TKY	TKY31	0.0166	0.25	0.0042	0	3	0.5009	0.83	0.4174	2	10
Turkey	Yagmurlar Koyu, Kutaha, TKY	TKY32	0.0108	0.25	0.0027	0	3	0.5873	1.00	0.5873	3	12
Turkey	Eskigomu Koyu, Afyonkarahisar, TKY	TKY33	0.0107	0.33	0.0035	0	4	0.5717	1.00	0.5717	3	12
Turkey	Adakoy Koyu, Zonguldak, TKY	TKY34	0.1219	0.42	0.0512	1	5	0.5667	1.00	0.5667	3	12
Turkey	Yonkali Koyu, Corum, TKY	TKY35	0.0231	0.25	0.0058	0	3	0.5533	1.00	0.5533	3	12
Turkey	Gundogan Mahallesi, Ankara, TKY	TKY36	0.0140	0.17	0.0024	0	2	0.5489	1.00	0.5489	3	12
Turkey	Dereli Koyu, Bartin, TKY	TKY37	0.4384	0.75	0.3288	3	9	0.5895	1.00	0.5895	3	12
Turkey	Ahlatcik Koyu, Corum, TKY	TKY38	0.0231	0.25	0.0058	0	3	0.5533	1.00	0.5533	3	12
Turkey	Yaylakent Koyu, Tokat, TKY	TKY39	0.0272	0.17	0.0046	0	2	0.5598	1.00	0.5598	3	12
Turkey	Akoren, Mengen, TKY	TKY40	0.2740	0.83	0.2274	2	10	0.5132	0.83	0.4277	2	10
	**Proportion of sites with infection index**	**0.983**										
**Southwest Asia (50)**									0.00			
Egypt	Unnamed, Hala'ib Triangle, EGY	EGY1	0	0.00	0.0000	0	0	0.0056	0.33	0.0019	0	4
Egypt	Unnamed, Qesm Marsa Alam, EGY	EGY2	0	0.00	0.0000	0	0	0.0297	0.50	0.0148	0	6
Egypt	Qism ras Ghareb, Red Sea, EGY	EGY3	0	0.00	0.0000	0	0	0.0763	0.50	0.0381	0	6
Egypt	Qesm Nwebaa, South Sinai, EGY	EGY4	0.0007	0.08	0.0001	0	1	0.2034	0.75	0.1526	1	9
Egypt	Arish, North Sinai, EGY	EGY5	0.0050	0.25	0.0013	0	3	0.1311	0.67	0.0874	0	8
Egypt	Kom-El-Farag, El Beheira, EGY	EGY6	0.0019	0.08	0.0002	0	1	0.0913	0.58	0.0533	0	7

Region and Country	Location	Code	*Pgt* MINF (B)	*Pgt* Fav-month (F)	*Pgt* Favourable index (B x F)	*Pgt* Risk score	*Pgt* Month	Barberry NHTT (D)	Barberry Fav-month (F)	Barberry Favourable growth index (D x F)	Barberry Suitabilty score	Barberry Months
Egypt	Qism Moury Matrouch, Matrough, EGY	EGY7	0.0100	0.17	0.0017	0	2	0.1202	0.67	0.0801	0	8
Egypt	Qesm Sidi Brani, Matrough, EGY	EGY8	0.3653	0.58	0.2119	2	7	0.0409	0.58	0.0238	0	7
Egypt	Qesm Sidi Brani 1, Matrough, EGY	EGY9	0.0004	0.08	0.0000	0	1	0.1276	0.67	0.0851	0	8
Egypt	Qesm Al Wahat Al Khargah 1, New Valley, E	EGY10	0	0.00	0.0000	0	0	0.1512	0.67	0.1008	1	8
Egypt	Qesm Al Wahat Al Khargah 2, New Valley, E	EGY11	0	0.00	0.0000	0	0	0.0690	0.50	0.0345	0	6
Egypt	Abu ar Rish Basri, Red Sea, EGY	EGY12	0	0.00	0.0000	0	0	0.0885	0.50	0.0442	0	6
Egypt	Qesm Al Wahat Ad Dakhlah 1, New Valley, E	EGY13	0	0.00	0.0000	0	0	0.1089	0.58	0.0635	0	7
Egypt	Qesm Hurghada 1, Red Sea, EGY	EGY14	0	0.00	0.0000	0	0	0.1072	0.58	0.0625	0	7
Egypt	Qesm Sham Ash Sheikh, South Sinai, EGY,	EGY15	0.0009	0.08	0.0001	0	1	0.0477	0.50	0.0238	0	6
Egypt	Ataqah 1, Suez, EGY	EGY16	0.0029	0.08	0.0002	0	1	0.1285	0.67	0.0857	0	8
Egypt	Qesm Hurghada 2, Red Sea, EGY	EGY17	0	0.00	0.0000	0	0	0.1063	0.58	0.0620	0	7
Egypt	Unanmed, Red Sea, EGY	EGY18	0	0.00	0.0000	0	0	0.1510	0.67	0.1007	1	8
Egypt	Ataqah 2, Suez, EGY	EGY19	0.0029	0.08	0.0002	0	1	0.1285	0.67	0.0857	0	8
Egypt	Shata, Damietta, EGY	EGY20	0.0203	0.08	0.0016	0	1	0.0481	0.58	0.0281	0	7
Iraq	Aghajah Mashad, Tooz, IQ	IRQ1	0.0275	0.33	0.0091	1	4	0.3229	0.67	0.2153	1	8
Iraq	Unnamed, Ana, IQ	IRQ2	0.0016	0.08	0.0001	0	1	0.2877	0.67	0.1918	1	8
Iraq	Unnamed, RTE France, KT	IRQ3	0	0.00	0.0000	0	0		0.50	0.0000	0	6
Iraq	Unnamed, Najaf, IQ	IRQ4	0	0.08	0.0000	0	1	0.2015	0.58	0.1175	1	7
Iraq	Unnamed, Ramadi, IQ	IRQ5	0.0055	0.08	0.0004	0	1	0.2279	0.58	0.1329	1	7
Israel	Givat Nili, Har Alexander, IS	IS1	0.1143	0.50	0.0571	1	6	0.1223	0.58	0.0713	0	7
Israel	Ohad, Eshkol, IS	IS2	0.0358	0.42	0.0151	1	5	0.0801	0.58	0.0467	0	7
Lebanon	Mazraat Matariyet Jbaa, Maghdouche', LB	Lb1	0.9615	0.75	0.7211	4	9	0.0723	0.58	0.0422	0	7
Oman	Fajri, Al Batinah, OM	OM1	0	0.00	0.0000	0	0	0.0216	0.42	0.0090	0	5
Oman	Unnamed, Ash Sharqiyah, South, OM	OM2	0	0.00	0.0000	0	0	0.0006	0.00	0.0000	0	0
Oman	Unnamed, Dhofar, OM	OM3	0	0.00	0.0000	0	0	0.0072	0.33	0.0024	0	4
Oman	Hai;at La'mia, Al Wusta, OM	OM4	0	0.00	0.0000	0	0	0.0156	0.33	0.0052	0	4
Saudi Arab	Tawala, Tabuk, SA	SA1	0	0.00	0.0000	0	0	0.0083	0.33	0.0028	0	4
Saudi Arab	Unnamed 1, Tabuk, SA	SA2	0.0009	0.08	0.0001	0	1	0.0719	0.50	0.0359	0	6
Saudi Arab	Unnamed 2, Tabuk, SA	SA3	0	0.00	0.0000	0	0	0.0184	0.42	0.0077	0	5

Region and Country	Location	Code	*Pgt* MINF (B)	Fav-month (F)	Favourable index (B x F)	Risk score	Month	Barberry NHTT (D)	Fav-month (F)	Favourable growth index (D x F)	Suitabilty score	Months
Saudi Arab	Unnamed, Al Qunfudhah, SA	SA4	0	0.00	0.0000	0	0	0.0017	0.17	0.0003	0	2
Saudi Arab	Shuwaq, Tabuk, SA	SA5	0	0.00	0.0000	0	0	0.0450	0.50	0.0225	0	6
Saudi Arab	Unanmmed, Forest area, SA	SA6	0	0.00	0.0000	0	0	0.2875	0.67	0.1917	1	8
Saudi Arab	Unnamed, Northern Borders, SA	SA7	0	0.00	0.0000	0	0	0.2323	0.58	0.1355	1	7
Saudi Arab	Unanmed, Al Nufud Al Kabir, SA	SA8	0	0.00	0.0000	0	0	0.1347	0.50	0.0674	0	6
Saudi Arab	Unnamed, Al Aflaj, SA	SA9	0	0.00	0.0000	0	0	0.0509	0.42	0.0212	0	5
Saudi Arab	Al Shinayin, Abu Dhabi, UAE	SA10	0	0.00	0.0000	0	0	0.0127	0.33	0.0042	0	4
Saudi Arab	Unnamed, Al Ahsa, SA	SA11	0.0146	0.25	0.0037	0	3	0.0388	0.42	0.0161	0	5
Syria	Babdh, Jableh, SY	SY1	0.0780	0.42	0.0327	1	5	0.2822	0.75	0.2117	1	9
Syria	Khirbet Ghazaleh, Daraa, SY	SY2	0.0409	0.25	0.0102	1	3	0.3193	0.83	0.2661	1	10
Syria	Palmyra, SY	SY3	0.0409	0.25	0.0102	1	3	0.3804	0.83	0.3170	2	10
Yemen	Karad, Ad Dali, YM	YM1	0	0.00	0.0000	0	0	0.0284	0.58	0.0165	0	7
Yemen	Samarma, Al Mahrah, YM	YM2	0	0.00	0.0000	0	0	0.0033	0.25	0.0008	0	3
Yemen	Unnamed, Hadhramaut, YM	YM3	0.0004	0.08	0.0000	0	1	0.0371	0.42	0.0154	0	5
Yemen	Al Harajah, Hajjah, YM	YM4	0	0.00	0.0000	0	0	0.0016	0.00	0.0049	0	4
	**Proportion of sites with infection index**	**0.24**										
**Adjoining European countries (53)**									0.00			
Albania	Sheshi, Tepelene, ALB	ALB1	0.7956	0.92	0.7320	4	11	0.3760	0.83	0.3133	2	10
Bulgaria	Zhitosvyat, Karnobat, BL	BL1	0.0294	0.25	0.0073	0	3	0.5125	1.00	0.5125	3	12
Bulgaria	Krupen, Kavarna, BL	BL2	0.1199	0.50	0.0599	1	6	0.5571	0.83	0.4643	2	10
Bulgaria	Prismenovo, Primorsko, BL	BL3	0.3110	0.83	0.2582	2	10	0.5536	0.83	0.4613	2	10
Bulgaria	Kartipanya, Dryanovo, BL	BL4	0.0748	0.42	0.0314	1	5	0.5355	1.00	0.5355	3	12
Bulgaria	Manastirishte, Hayredin, BL	BL5	0.1020	0.42	0.0428	1	5	0.4976	1.00	0.4976	2	12
Bulgaria	Pomen, Dve mongili, BL	BL6	0.0741	0.42	0.0311	1	5	0.5129	1.00	0.5129	3	12
Bulgaria	Byalkovtsi, Elena, BL	BL7	0.0418	0.25	0.0105	1	3	0.5278	1.00	0.5278	3	12
Bulgaria	Trakia, Opan, BL	BL8	0.0506	0.25	0.0127	1	3	0.5191	1.00	0.5191	3	12
Bulgaria	Byal Kladenets, Stambolovo, BL	BL9	0.0396	0.25	0.0099	1	3	0.5099	1.00	0.5099	3	12
Bulgaria	Lyahovo, Balchik, BL	BL10	0.5250	0.83	0.4357	3	10	0.5896	0.83	0.4913	2	10

			Pgt					Barberry				
Region and Country	Location	Code	MINF (B)	Fav-month (F)	Favourable index (B x F)	Risk score	Month	NHTT (D)	Fav-month (F)	Favourable growth index (D x F)	Suitabilty score	Months
Croatia	Ridane, CR	CR1	0.4811	1.00	0.4811	3	12	0.5578	1.00	0.5578	3	12
Croatia	Baska, Opcina, CR	CR2	0.8308	1.00	0.8308	4	12	0.8407	1.00	0.8407	4	12
Greece	Doumpia, Anthemountas, GR	GR1	0.1527	0.83	0.1268	2	10	0.5022	0.83	0.4185	2	10
Greece	Kokkinaras, Koropi, GR	GR2	0.8089	0.83	0.6714	3	10	0.1736	0.67	0.1158	1	8
Greece	Gergeri, Rouvas, GR	GR3	1.0000	0.83	0.8300	4	10	0.1047	0.67	0.0698	0	8
Greece	Dafniotissa, Amaliada, GR	GR4	0.9090	0.83	0.7545	4	10	0.1142	0.67	0.0761	0	8
Greece	Plagia, Acheloos, GR	GR5	0	0.00	0.0000	0	0	1.0000	1.00	1.0000	4	12
Greece	Paleochori, Sperchiada, GR	GR6	0.2358	0.75	0.1768	2	9	0.5426	0.83	0.4522	2	10
Italy	Ai Boschi Castelminio, Treviso, IT	IT1	0.3172	1.00	0.3172	3	12	0.6205	1.00	0.6205	3	12
Italy	Ostellato, Ferrara, IT	IT2	0.1734	0.83	0.1439	2	10	0.5249	0.83	0.4374	2	10
Italy	Sassocorvaro, Pesaro, IT	IT3	0.2519	0.92	0.2318	2	11	0.5932	1.00	0.5932	3	12
Italy	Campli, Teramo, IT	IT4	0.6462	1.00	0.6462	3	12	0.5553	0.83	0.4627	2	10
Italy	Acquaviva Collecroce, Campobasso, IT	IT5	0.4772	0.83	0.3961	3	10	0.8407	1.00	0.8407	4	12
Montenegro	Lipa, Cetinje, MN	MN1	0.3558	0.92	0.3274	3	11	0.6800	1.00	0.6800	3	12
Romania	Unnamed, Casimcea, RM	RM1	0.0515	0.25	0.0129	1	3	0.5281	0.92	0.4841	2	11
Romania	Valea Seman, Urlati, RM	RM2	0.0437	0.42	0.0184	1	5	0.5517	1.00	0.5517	3	12
Romania	Maneciu-Ungureni, Maneciu, RM	RM3	0.0437	0.42	0.0184	1	5	0.5517	1.00	0.5517	3	12
Romania	Unnamed, Bustuchin, RM	RM4	0.0886	0.42	0.0372	1	5	0.5477	1.00	0.5477	3	12
Romania	Piscu Nou, Piscu Vechi, RM	RM5	0.1071	0.42	0.0450	1	5	0.5029	1.00	0.5029	3	12
Romania	Valcelele de Sus, Valcele, RM	RM6	0.0591	0.42	0.0248	1	5	0.5096	1.00	0.5096	3	12
Romania	Runc, Zlatna, RM	RM7	0.1476	0.50	0.0738	1	6	0.5677	1.00	0.5677	3	12
Romania	Strugureni, Chiochis, RM	RM8	0.0816	0.50	0.0408	1	6	0.5501	1.00	0.5501	3	12
Romania	Cuza Voda, racovita, RM	RM9	0.0464	0.42	0.0195	1	5	0.5170	1.00	0.5170	3	12
Romania	Razim lake, RM	RM10	0.5397	0.42	0.2267	2	5	0.5344	1.00	0.5344	3	12
Romania	Podul Lung, Calarasi, MLD	RM11	0.0321	0.83	0.0266	1	10	0.6056	0.83	0.5047	3	10
Romania	Caltesti, Pietroasele, RM	RM12	0.0448	0.33	0.0148	1	4	0.5005	1.00	0.5005	3	12
Slovenia	Vedrijan, SL	SL1	0.2465	0.67	0.1651	2	8	0.6700	1.00	0.6700	3	12
Ukraine	Verkhni Stanivtsi, Chernivtsi Oblast, UKR	UKR1	0.0793	0.33	0.0262	1	4	0.6323	0.83	0.5269	3	10
Ukraine	Pavlivka, Odessa Oblast, UKR	UKR2	0.1401	0.50	0.0701	1	6	0.5669	0.83	0.4724	2	10

Region and Country	Location	Code	*Pgt* MINF (B)	Fav-month (F)	Favourable index (B x F)	Risk score	Month	Barberry NHTT (D)	Fav-month (F)	Favourable growth index (D x F)	Suitabilty score	Months
Ukraine	Creamian Peninsula, UKR	UKR3	0.2909	0.67	0.1949	2	8	0.5416	1.00	0.5416	3	12
Ukraine	Sevastopol 1, Crimean, UKR	UKR4	0.0854	0.50	0.0427	1	6	0.5770	0.83	0.4808	2	10
Ukraine	Sevastopol 2, Crimean, UKR	UKR5	0.1597	0.58	0.0926	1	7	0.5900	0.83	0.4917	2	10
Ukraine	Novohryhorivka, Kherson Oblast, UKR	UKR6	0.5046	0.83	0.4188	3	10	0.6231	0.75	0.4673	2	9
Ukraine	Yelyseivka, Zaporizhia Oblast, UKR	UKR7	0.2682	0.75	0.2011	2	9	0.5855	0.83	0.4879	2	10
Ukraine	Ahronomichne, Donetsk Oblast, UKR	UKR8	0.0530	0.33	0.0175	1	4	0.4730	1.00	0.4730	2	12
Ukraine	Shtormove, Luhansk Oblast, UKR	UKR9	0.0448	0.50	0.0224	1	6	0.4157	1.00	0.4157	2	12
Ukraine	Zavadivka, Kirovohrad Oblast, UKR	UKR10	0.0480	0.42	0.0202	1	5	0.4775	1.00	0.4775	2	12
Ukraine	Kunyche, Vinnytsia Oblast, UKR	UKR11	0.0280	0.33	0.0092	1	4	0.4850	1.00	0.4850	2	12
Ukraine	Potaptsi, Cherkasy Oblast, UKR	UKR12	0.0911	0.42	0.0382	1	5	0.4186	1.00	0.4186	2	12
Ukraine	Kalivka, Sumy Oblast, UKR	UKR13	0.0837	0.42	0.0351	1	5	0.4616	1.00	0.4616	2	12
Ukraine	Rohovchi, Khmelnytskyi Oblast, UKR	UKR14	0.0624	0.50	0.0312	1	6	0.5014	1.00	0.5014	3	12
Ukraine	Novi Sokoly, Kyiv Oblast, UKR	UKR15	0.0906	0.50	0.0453	1	6	0.0927	1.00	0.0927	0	12
	**Total number of sites or *mean***	**607**	***0.1099***	***0.3305***	***0.0679***	***####***	***3.97***	***0.3974***	***0.8129***	***0.3608***	***1.7957***	***9.8616***

Table S2. Numbers and percentages of locations in Asian and Southwest European regions with different stripe rust infection suitability scores.

Region (locations)	Number (%) of sites with different infection score (*MINF* × *F*)[a]					
	0	1	2	3	4	1 to 4
East Asia (250)	63(25.2*)	163(65.2)	24(9.6)	0	0	187(74.8))
Central Asia (195)	54(27.7)	114(58.5)	15(7.7)	10(5.1)	2(1.0)	141(72.3))
Northwest Asia (59)	1(1.7)	42(71.2)	6(10.2)	7(11.9)	3(5.1)	58(98.3)
Southwest Asia (50)	40(80)	9(18)	0	1(2)	0	10(20.0)
Southeast Europe (53)	0	32(60.4)	10(18.9)	10(18.9)	1(1.9)	53(100.0)
Total (607)	158(26.0)	360(59.3)	55(0.09)	28(0.05)	6(0.01)	449(74.0)

[a] *MINF* = normalized mean monthly infection index; *F* = Fav-month; Scores: 0 = 0 to 0.0080; 1 = 0.0081 to 0.1000; 2 = 0.1001 to 0.3000; 3 = 0.3001 to 0.7000; and 4 = 0.7001 to 1.0.

Table S3. Numbers and percentages of locations in Asian and Southwest European regions with different stem rust infection suitability scores.

Region (locations)	Number (%) of sites with different infection score ($MINF \times F$)[a]					
	0	1	2	3	4	1 to 4
East Asia (250)	61(24.4)	35(14)	82(32.8)	68(27.2)	4(1.6)	189(75.6)
Central Asia (195)	54(27.7)	21(10.8)	48(24.6)	58(29.7)	14(7.2)	141(72.3)
Northwest Asia (59)	1(1.7)	7(11.9)	26(44.1)	18(30.5)	7(11.9)	58(98.3)
Southwest Asia (50)	38(76)	5(10)	4(8)	3(6)	0	12(24.0)
Southeast Europe (53)	0	0	6(11.3)	33(62.3)	14(26.4)	53(100.0)
Total (607)	154(25.4)	68(11.2)	166(27.3)	180((29.7)	39(0.06)	453(74.6)

[a] *MINF* = normalized mean monthly infection index; *F* = Fav-month; Scores: 0 = 0 to 0.0080; 1 = 0.0081 to 0.1000; 2 = 0.1001 to 0.3000; 3 = 0.3001 to 0.7000; and 4 = 0.7001 to 1.0.

Table S4. Numbers and percentages of locations in Asian and Southwest European regions with different barberry growth suitability scores.

Region (locations)	Number (%) of sites with different growth score (*NHTT* x *F*)[a]					
	0	1	2	3	4	1 to 4
East Asia (250)	57(22.8)	43(17.2)	84(33.6)	45(18.0)	21(8.4)	193(77.2)
Central Asia (195)	9(4.6)	50(25.6)	85(43.6)	49(25.1)	2(1.1)	186(95.4)
Northwest Asia (59)	2(3.4)	7(11.9)	15(25.4)	35(59.3)	0	57(96.6)
Southwest Asia (50)	38(76)	11(22)	1(2)	0	0	12(24.0)
Southeast Europe (53)	3(5.7)	1(1.9)	21(39.6)	25(47.2)	3(5.7)	50(94.3)
Total (607)	109(18.0)	112(18.5)	206(33.9)	154(25.4)	26(4.3)	498(82.0)

[a] *MINF* = normalized mean monthly infection index; *F* = Fav-month; Scores: 0 = 0 to 0.0080; 1 = 0.0081 to 0.1000; 2 = 0.1001 to 0.3000; 3 = 0.3001 to 0.7000; and 4 = 0.7001 to 1.0.

Table S5. Scores of infections of *Puccinia striiformis* f. sp. *tritici* (*Pst*) and *P. graminis* f. sp. *tritici* (*Pgt*) on barberry and barberry growth predicted using the framework of this study and the Dymex model for selected locations in the U.S. Pacific and western China with reported barberry growth and the correlation of the two model results determined using Cohen's Kappa coefficient (*k*).

					Infection or growth score					
					Framework			Dymex model		
Location	Code	Lat°N	Long°W	Elev (m)	*Pst*	*Pgt*	Barberry	*Pst*	*Pgt*	Barberry
**U.S. Pacific**					**k = 0.68 (*Pst*); 0.50 (*Pgt*); 0.50 (barberry)**					
Red Sport, OR	US3	43.7018	-123.8298	244.9	3	4	4	3	4	4
Cox Bar, CA	US4	40.9738	-123.1267	1027.5	2	1	4	3	2	4
Cle-Elm-Roslyn, WA	US5	47.1544	-121.2810	1199.3	2	2	4	2	2	2
Hood Canal, WA	US6	47.5711	-123.3903	500.9	4	4	4	4	4	4
Bend, OR	US7	43.8604	-120.9734	1378.8	3	3	3	3	4	4
Lake County, OR	US8	42.3851	-120.7976	1609.9	2	2	3	2	3	2
Big Creek, CA	US9	37.5382	-118.9519	2433.4	0	0	3	0	1	0
Whitman, WA	US10	46.9401	-117.5264	630.7	0	0	3	1	0	4
Ellensburg, WA	US11	46.9846	-120.5263	869.1	0	0	3	1	1	3
Lincoln, WA	US12	47.5428	-118.5969	664.7	0	0	3	1	0	4
**Western China**					**k = 0.68 (*Pst*); 0.56 (*Pgt*); 0.40 (barberry)**					
Aba, Sichuan	CH106	31.8975	102.2082	3830.1	0	0	3	0	0	2
Baoji-1, Shaanxi	CH107	34.3787	107.1298	1368.9	0	0	3	1	1	1
Bazhong, Sichuan	CH108	31.8666	106.7181	597.9	0	2	2	0	2	2
Chengxian, Gansu	CH109	33.7436	105.6880	1489.3	0	0	3	0	1	2
Dali, Yunnan	CH110	25.6058	100.1786	2212.9	0	0	3	0	1	3
Degen, Yunnan	CH111	24.1786	100.7496	1714.4	0	0	2	1	0	2
Deyang, Sichuan	CH112	31.1405	104.3301	979.2	1	2	3	1	2	3
Dinxi, Gansu	CH113	35.5888	104.6410	1982.8	0	0	3	0	0	2
Fengxian, Shaanxi	CH114	33.9258	106.5501	1444.4	0	0	3	0	1	2
Gangu, Gansu	CH115	34.7232	105.3472	1852.8	0	0	3	1	0	3
Gannan, Gansu	CH116	36.4891	103.6229	2022.9	0	0	3	0	1	3
Ganzi, Sichuan	CH117	30.0683	101.9874	3897.4	0	0	3	0	1	2
Guangyuan, Sichuan	CH118	32.4557	105.4557	845.9	0	2	3	1	2	3
Haixi, Qinghai	CH119	37.3859	97.3790	3362.6	0	0	3	0	1	1
Hanzhong, Shaanxi	CH120	33.0695	106.9625	1140.6	2	0	3	2	0	2
Huixian, Gansu	CH121	33.7688	106.0569	1402.6	0	0	3	0	1	3
Lueyang, Shaanxi	CH122	34.6303	107.1306	1249.6	0	0	3	1	1	2
Kunming, Yunan	CH123	24.9522	102.8935	1938.5	0	0	2	0	0	1
Lanzhou, Gansu	CH124	36.4891	103.6229	2022.9	0	0	3	1	0	2
Liangdang, Gansu	CH125	33.9050	106.2660	1402.6	0	0	3	0	0	3

Aba, Sichuan	CH106	31.8975	102.2082	3830.1	0	0	3	0	0	3
Baoji-2, Shaanxi	CH107	34.3787	107.1298	1368.9	0	0	3	2	1	2
Bazhong, Sichuan	CH108	31.8666	106.7181	597.9	0	2	3	0	2	2
Chengxian, Gansu	CH109	33.7436	105.6880	1489.3	0	2	3	0	2	3
Dali, Yunnan	CH110	25.6058	100.1786	2212.9	0	0	3	1	1	2

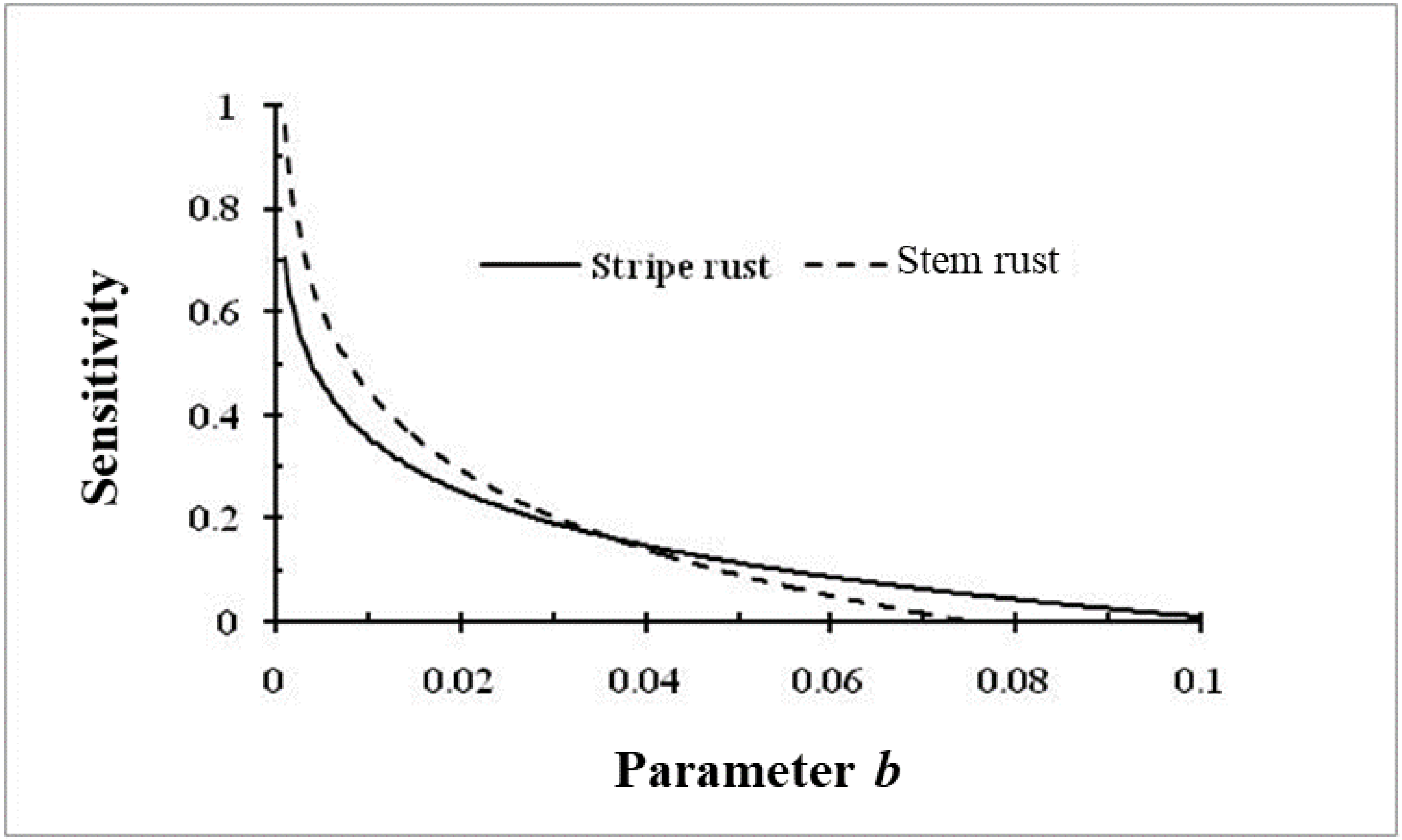

1
0.8
0.6
0.4
0.2
0
Sensitivity
Stripe rust
Stem rust
0
0.02
0.04
0.06
0.08
0.1
Parameter b

Fig. S1

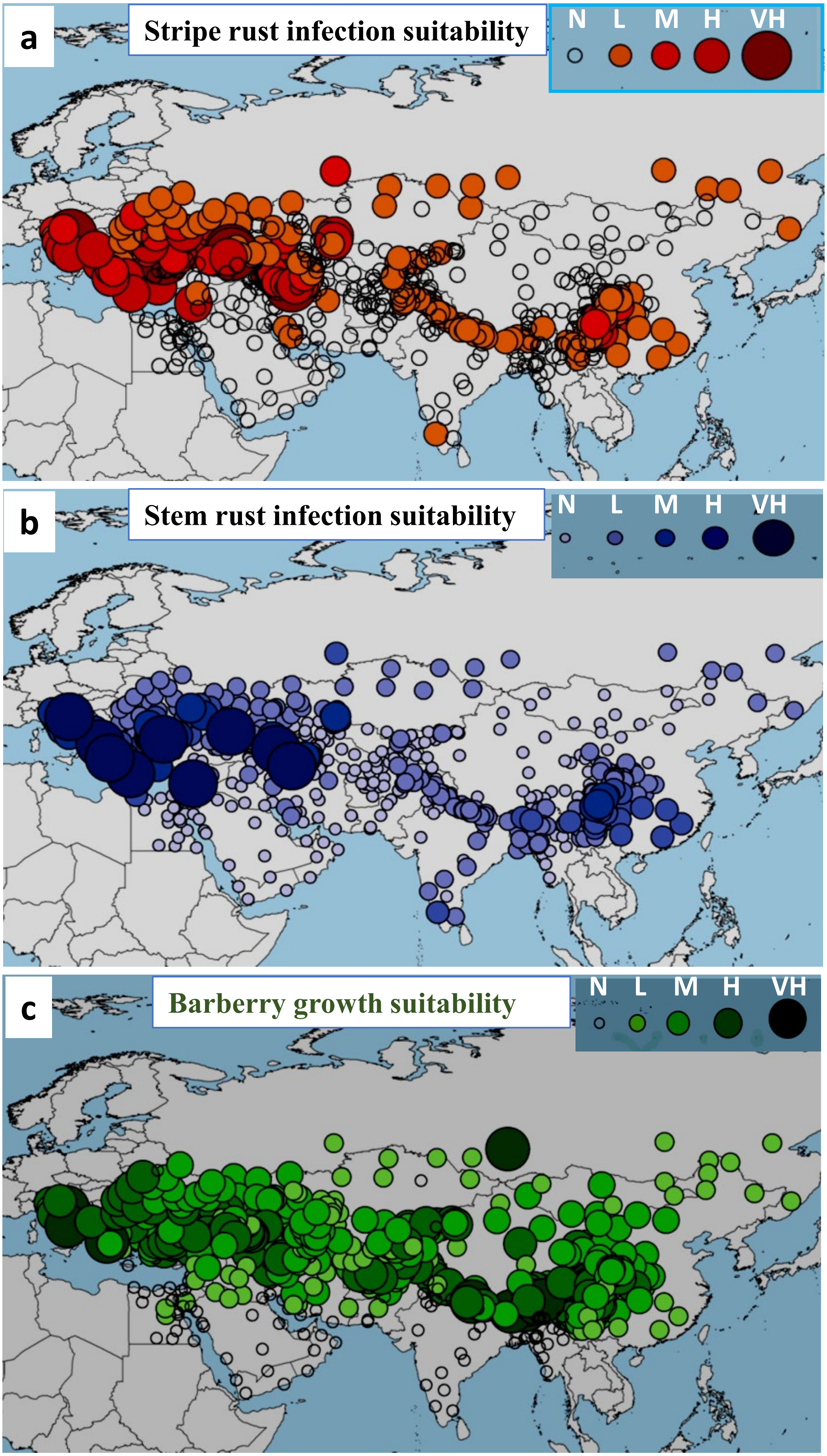
a
Stripe rust infection suitability
N L M H VH
b
Stem rust infection suitability
N L M H VH
c
Barberry growth suitability
N L M H VH

Fig. S2

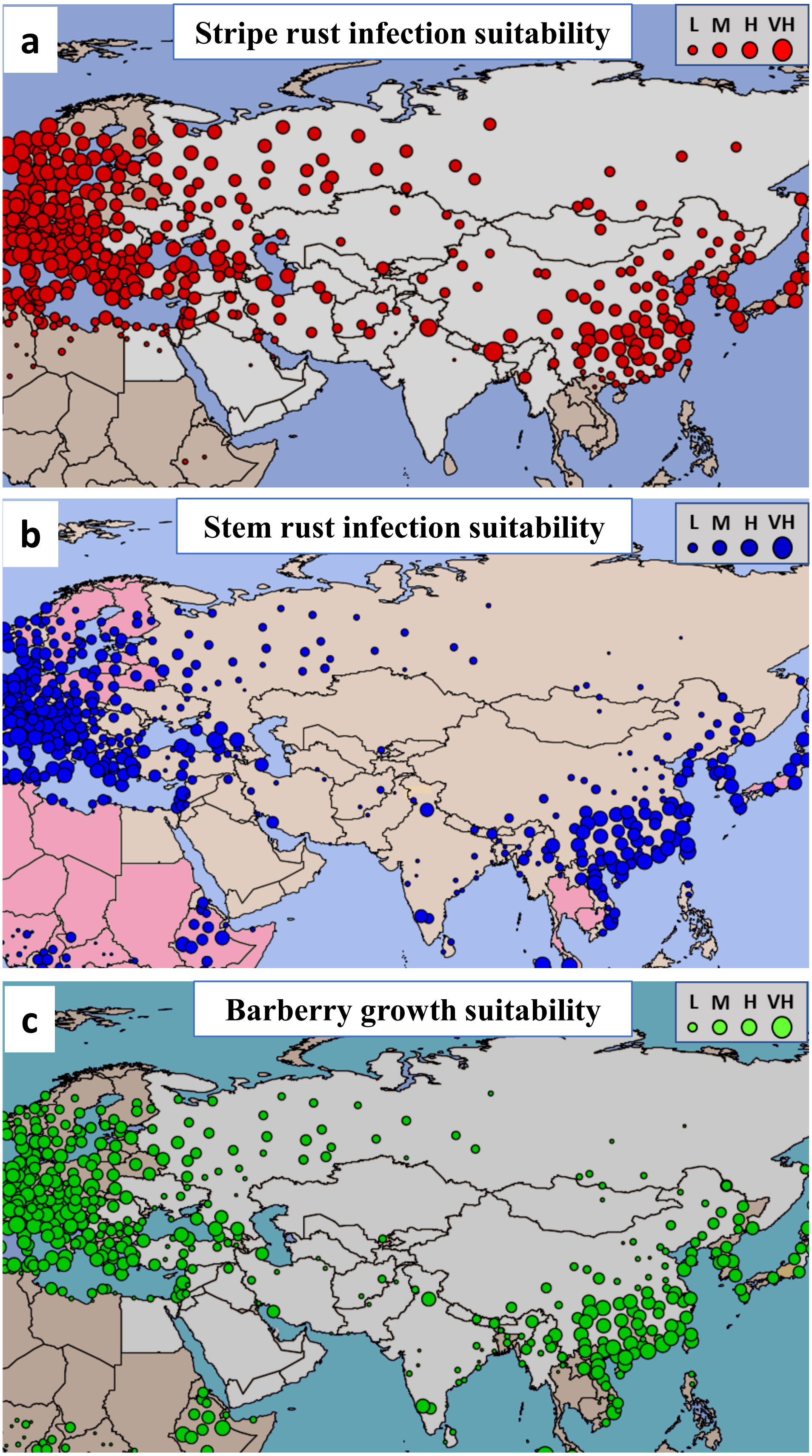
a
Stripe rust infection suitability
L M H VH
b
Stem rust infection suitability
L M H VH
c
Barberry growth suitability
L M H VH

Fig. S3
